# Designing Effective and Acceptable Road Pricing Schemes: Evidence from the Geneva Congestion Charge

**DOI:** 10.1007/s10640-021-00564-y

**Published:** 2021-05-31

**Authors:** Andrea Baranzini, Stefano Carattini, Linda Tesauro

**Affiliations:** 1 Haute école de gestion de Genève, HEG-Genève, University of Applied Sciences and Arts Western Switzerland, HES-SO, Geneva, Switzerland; 2 Department of Economics, Andrew Young School of Policy Studies, Georgia State University, Atlanta, USA; 3 CESifo, Munich, Germany; 4 Grantham Research Institute on Climate Change and the Environment and ESRC Centre for Climate Change Economics and Policy, London School of Economics and Political Science, London, UK; 5 SIAW-HSG and Department of Economics, University of St. Gallen, St. Gallen, Switzerland; 6 Laboratory of Environmental and Urban Economics (LEURE), Ecole polytechnique fédérale de Lausanne (EPFL), Lausanne, Switzerland

**Keywords:** Acceptability, Congestion charge, Policy design, Public support, Road pricing, D72, H23, Q53, Q58, R41, R48

## Abstract

While instruments to price congestion exist since the 1970s, less than a dozen cities around the world have a cordon or zone pricing scheme. Geneva, Switzerland, may be soon joining them. This paper builds on a detailed review of the existing schemes to identify a set of plausible design options for the Geneva congestion charge. In turn, it analyzes their acceptability, leveraging a large survey of residents of both Geneva and the surrounding areas of Switzerland and France. Our original approach combines a discrete choice experiment with randomized informational treatments. We consider an extensive set of attributes, such as perimeter, price and price modulation, use of revenues, and exemption levels and beneficiaries. The informational treatments address potential biased beliefs concerning the charge’s expected effects on congestion and pollution. We find that public support depends crucially on the policy design. We identify an important demand for exemptions, which, albeit frequently used in the design of environmental taxation, is underexplored in the analysis of public support. This demand for exemptions is not motivated by efficiency reasons. It comes mostly by local residents, for local residents. Further, people show a marked preference for constant prices, even if efficiency would point to dynamic pricing based on external costs. Hence, we highlight a clear trade-off between efficiency and acceptability. However, we also show, causally, that this gap can in part be closed, with information provision. Analyzing heterogeneity, we show that preferences vary substantially with where people live and how they commute. Even so, we identify several designs that reach majority support.

## Introduction

Traffic congestion is among the top issues in many cities in the world. Congestion generates important costs to society, due to extended journeys, local and global pollution, noise, and accidents. Over the next few decades, projections predict a large increase in urban population, in both developed and developing countries (United Nations 2015), potentially increasing total motorized mobility by about 40% by 2030 with respect to 2015, and by over 90% by 2050 (OECD/ITF 2017). However, the extent to which an increasing urban population translates into higher traffic congestion depends on public policy. Interest in curbing traffic congestion and reducing local air pollution has likely increased following the recent COVID-19 lockdowns, which made very salient the contribution of motorized traffic to air pollution and other exernal costs (Berman and Ebisu 2020; Cicala et al. 2020; He et al. 2020; Muhammad et al. 2020). Congestion is one of the classic textbook examples of an externality, whose economic theory dates back to Pigou (1920). Time lost in traffic is the main externality from traffic congestion (Small et al. 2005, Table 3.3; Small et al. 2007), followed by car accidents and pollution among others (e.g. Li et al. 2012; Jacobsen 2013). The solution to the external costs of driving is well known since the 1960s: pricing road traffic (Walters 1961; Reynolds 1963; Vickrey 1963). However, very few jurisdictions in the world have implemented congestion charges. Unlike the climate externality, traffic congestion is a very local issue, and intergenerational equity issues are largely absent. Yet, there are only a handful of cities in the world with a cordon or zone pricing scheme, compared to some 60 jurisdictions pricing carbon (World Bank 2020). Hence, the 1963 statement by William S. Vickrey, that “in no other major area are pricing practices so irrational, so out of date, and so conducive to waste as in urban transportation” is even more relevant today than it was in the 60s (Vickrey 1963, p. 452).

The main reason for the extremely slow emergence of congestion charges in world cities is arguably lack of public support (Gu et al. 2018). Policymakers in many cities, including New York, have in the past abandoned their plans for a congestion charge due to lack of public support. A congestion charge was rejected at the ballot box in Birmingham, Edinburgh, and Manchester. Voters in Gothenburg rejected it in a referendum, although the scheme was nevertheless implemented given the non-binding character of the referendum. Voters in Stockholm approved it, but only after a trial period.

Standard political economy theory shows that even policies that are clearly beneficial for society may not actually be implemented, mainly because of the mismatch between policymakers’ incentive to be reelected and society’s welfare (e.g. Dixit et al. 1997; Samuelson and Zeckhauser 1988; Hahn 1989; Maskin and Tirole 2004). However, reforms may not only create discontent among losers, but potentially also among winners, if the latter do not correctly anticipate, ex ante, the benefits of the policy change (Kallbekken et al. 2011; Dal Bó et al. 2018). Winners may also have preferences towards fairness, related to both the polluter-pay principle and distributional effects. Equity issues may thus be in conflict with efficient congestion charge designs (see Kristoffersson et al. 2017), which implies that the more efficient designs proposed in economic theory could be less accepted by the population.

The federal government of Switzerland recently encouraged cantons to consider mobility pricing, a dynamic pricing approach to mobility that includes the use of congestion charges, to tackle congestion on roads as well as other transportation modes. Despite the high levels of congestion in Swiss cities, not many cantons stepped forward. In a country where public ballots take place about 4 times a year on multiple issues, the political stakes are very high. The Canton of Geneva, however, did so, launching a policy process aimed at identifying the best congestion charge design for the city of Geneva. Geneva is the most congested city in Switzerland, with about 2,000,000 trips per day in the agglomeration in 2010, at an average speed of about 20 km per hour in the city center (DETA 2014). Geneva has struggled for years to find solutions to its ever increasing traffic.

The aim of this paper is twofold. First, it builds on the existing theoretical and empirical literature on congestion charges to identify a set of plausible designs that could fit the context of Geneva. Second, it tackles the issue of public support related to the implementation of environmental policy instruments. It uses a large survey of respondents in both the Canton of Geneva and the neighboring regions of Switzerland and France to assess public support for the policy designs identified in this study. It relies on a discrete choice experiment to estimate preferences for the following parameters: charge rate, perimeter of the charge, modulation of the charge, level of exemptions, beneficiaries of exemptions (if any), and use of revenues. Given the large sample, and its cross-national feature, heterogeneity across respondents is analyzed in detail. Public support may also depend on the information available to respondents. A stylized fact, discussed in detail in the following sections, is that very salient environmental policies tend to be more popular ex post than ex ante. This finding may rationalize the fact that, in some contexts, congestion charges might have been implemented without majority support, while never being repealed (De Borger and Proost 2012). Specifically, people may revise their beliefs while experiencing their effectiveness (Cherry et al. 2014; Janusch et al. 2020). The acceptability of congestion charges increased after their implementation in Stockholm (Winslott-Hiselius et al. 2009; Eliasson 2014) and in London (Schade and Baum 2007). However, implementing a congestion charge, even if only for a trial period, implies considerable sunk costs and requires important political capital. Hence, providing more information to the general public, ex ante, may represent an effective alternative to trialing in increasing public support (see Carattini et al. 2017, 2019). In this paper, we go a step forward and explicitly test this hypothesis in a stated preference context, by coupling the discrete choice experiment with a split sample design introducing two randomized informational treatments, and a control group.

Our results show that public support depends closely on the design as well as the information provided, in particular with respect to the environmental benefits of a congestion charge. Public support decreases (increases) considerably with the charge rate (exemptions). However, the provision of information, especially on indirect benefits that may not be immediately factored in voters’ opinions, such as improved air quality, can increase public support and make more ambitious policies politically palatable. Providing information seems a relatively inexpensive strategy that could allow policymakers to push more stringent policies past the majority threshold. However, even so, concessions from the ideal of efficiency may be necessary. For instance, public support is stronger for exemptions to residents, rather than motorbikes or alternative fuel vehicles. Also, while on efficiency grounds congestion charging should match as closely as possible the marginal damage of driving, people tend to have a strong preference for a constant, predictable modulation. Similarly, most people demand earmarking for improvements in public transportation rather than a revenue-neutral approach. Finally, we identify substantial heterogeneity in our sample. Preferences for either a more compact perimeter or an extended area depend on where people live and how they commute. The same applies to spending, and exemptions. That is, public support can vary considerably depending on who has the right to say over the implementation of a congestion charge, in particular between residents of the charged area and people in the suburban areas around it.

This paper contributes to two strands of literature. First, it contributes to the literature on congestion charges, and road pricing more in general. Second, it contributes to the literature on public support for environmental policy. In this respect, the contribution is twofold. First, it provides evidence on people’s preferences for different design parameters and on their role for public support in a context wherein a congestion charge is an option under serious consideration. Its design also allows assessing the role of exemptions, whose effect on public support has been underexamined despite exemptions having been widely used not only for congestion charges but for environmental taxes at large, including carbon taxes (see World Bank 2020). Second, it provides a methodological contribution. It tackles the issue of information asymmetries with randomized informational treatments in combination with a discrete choice experiment. It shows, more in general, that hypotheses on the role of information on public support can be tested directly, with the simple use of randomized treatments.

The remainder of the paper is given as follows. Section 2 provides background information about congestion charges and institutional knowledge about the local context of this study. Section 3 describes the survey design and data. Section 4 provides the main empirical findings. Section 5 concludes.

## Background

### The External Costs of Driving

In the spirit of Pigou (1920), Vickrey (1963) suggested the implementation of pricing systems attributing to drivers the social cost of their driving, inclusive of the cost borne by the other commuters. Given the traffic externality, Pigouvian pricing should be introduced to make drivers pay for the (high) marginal cost of their use of street space. Vickrey’s analysis pointed to large welfare gains from road pricing, derived in particular by the change in traffic during peak hours, when the extra cost of an additional car is the highest, as the infrastructure capacity is pushed to the limit and the speed of other drivers is affected. Welfare gains of different road pricing designs have been estimated by several studies, including Walters (1961), Arnott et al. (1993), Parry (2002), and Yang et al. (2020).

Congestion may not be the only externality of driving. An emerging literature has recently developed, linking traffic, pollution, and health (see Currie et al. 2014 for a review). Following the implementation of electronic tolls in New Jersey and Pennsylvania, Currie and Walker (2011) find a decrease in nitrogen dioxide (NO_2_) and in the probability of prematurity and low-weight births by about 10% in the areas surrounding the tolls. Knittel et al. (2016) show with data for California that a standard deviation increase in traffic around a given area is related with a 0.2% standard deviation increase in mortality. Higher pollution levels are also related to higher infant mortality. Simeonova et al. (2018) find an immediate reduction in asthma among young children after the Stockholm congestion charge was initially trialed, but a much larger effect once it became permanent, pointing to the non-linear effects of exposure to pollution on health.

Recent work extends the analysis of the impacts of air pollution from traffic to adults. For instance, Zhong et al. (2017) show that in periods with higher traffic, emergency room visits in Beijing for fever and heart-related symptoms become substantially more frequent. Other health impacts related to air pollution include depressive symptoms (Zhang et al. 2017) and lower cognitive skills, in both the short and long run, such as productivity losses leading to lower test scores (Lavy et al. 2014) and lower lifetime earnings (Bharadwaj et al. 2017). The recent COVID-19 pandemic adds to this list. Contemporaneous exposure to fine particulate matter from various sources, including traffic, has been shown to have a substantial detrimental effect on COVID-19 morbidity and mortality (Austin et al. 2020).

Additionally, congestion charges may also have an effect on accidents, but this effect is a priori ambiguous. Lower congestion may decrease the risk of collision between cars and other road users, but may also imply higher speed and thus a higher likelihood of severe accidents (Shefer and Rietveld 1997). Cyclists and pedestrians are the most vulnerable road users, and their number tends to increase if people are incentivized not to use cars (Wang et al. 2009; Li et al. 2012). Also, congestion charges can potentially divert traffic to other, unpriced areas (Parry and Bento 2002). Green et al. (2016) conclude for the London congestion charge that its net effects on accidents and severe accidents are such that the congestion charge is beneficial.

### Existing Congestion Charges

Table 1 summarizes the characteristics of all cordon and zone pricing schemes currently in function. We are aware that other schemes to tackle traffic congestion directly or indirectly exists, such as tolled roads and bridges (e.g. Currie and Walker 2011), toll lanes (e.g. Bento et al. 2020) or smart-parking programs (e.g. Krishnamurthy and Ngo 2020). These road pricing options are related to our study, but do not inform directly its design.

**Table 1:** Summary of existing congestion charges schemes

	Stated goal	Perimeter	Type and price level	Modulation	Partial exemption	Use of revenue	References
Singapore (1975 and 1998)	Congestion	Center (central business district), 7 km^2^, and some highways	Zone, $0.5-$6 per passage through the gantry	7 am to 8 pm and by vehicle type, exact location	None		Khan (2001), Goh (2002), Agarwal and Koo (2016)
Bergen (1986)	Financial, environmental	Center, 18 km^2^	Cordon, NOK 19 to NOK 90 per way in	6.30 to 8.59 am and 2.30 to 4.59 pm Monday to Friday and by vehicle type (car vs. truck)	Seasonal passes (1, 3, 6, 12 months) and pre-paid passes (20 crossings)	Initially only for financing road projects, then 45% for road construction and 55% for improving environmental quality and road safety	Ramjerdi et al. (2004), Ieromonachou et al. (2006)
Oslo (1990)	Financial	Center, 64 km^2^	Cordon, NOK 40 to NOK 193 per way in	24/7 365 days a year, pricing by vehicle type (light vs. heavy and fuel type)	Seasonal passes (1, 6, 12 months) and pre-paid passes (25, 175, 350 crossings)	For investments in road capacity and public transportation projects	
Trondheim (1991)	Financial	Center, 24 km^2^ then 50 km^2^	Cordon, NOK 11 to NOK 72 per way in and hour depending on toll station	6 am to 6 pm Monday to Friday, and by vehicle type	Online payment	For financing road infrastructure (road capacity, with some earmaking to public transportation and to cycling and walking)	
London (2003)	Congestion	Center, 21km^2^	Zone, £11.50 per day	7 am to 6 pm Monday to Friday	By vehicle type (more than 9 seats, ultra low emissions, motorcycles) and resident status	For financing public transportation (80%), road safety (11%), cycling and walking (9%)	Leape (2006), Green et al. (2016), Tang (2016), Croci (2016)
Stockholm (permanent 2007)	Congestion	Center, 30 km^2^	Cordon, SEK 11 to SEK 35 per way in and out	6.30 am to 6.29 pm Monday to Friday, pricing depending on congestion levels (revised every 30 minutes)	None, but maximum SEK 105 per day	For financing road infrastructure and public transportation	Hensher and Li (2013), Croci (2016), Simeonova et al. (2018)
Milan (2008 and 2012)	Environmental, then congestion	Center, 8.2 km^2^	Cordon, € 5 per day when entering	7.30 am to 7.30 pm Monday to Friday and by vehicule type (Euro 0, 1, 2, 3)	By resident status, discounts for multiple days	For financing public transportation and cycling and walking	Percoco (2013), Hensher and Li (2013), Percoco (2014), Gibson and Carnovale (2015), Croci (2016)
Gothenburg (2013)	Congestion, environmental, financial	Center and highway	Cordon, SEK 9 to SEK 22 per way in and out	6 am to 6.29 pm Monday to Friday	None, but maximum SEK 60/day	For financing road infrastructure and public transportation	Börjesson and Kristoffersson (2015), Andersson and Nässén (2016)

The first congestion charge scheme was implemented in the central business district of Singapore in 1975. As a result, traffic entering the zone (circulating within the zone) decreased by 25% (45%) and travel speed doubled in the morning peaks (Khan 2001; Goh 2002). In 1990, the coverage was expanded to include a number of expressways. In 1998, the manual road pricing system was replaced, and Singapore became the first city to introduce electronic tolls. Thanks to the electronic system, drivers can be charged according to their vehicle type and their travel speed, which is used as a proxy for congestion.

After Singapore, several urban toll rings were implemented in Norway; in Bergen in 1986, in Oslo in 1990, in Trondheim in 1991, in Kristiansand in 1997, and in Stavanger in 2001. The toll rings of Bergen and Oslo were then converted to congestion charges in 2016 and 2017, respectively. The main objective of the Norwegian toll rings was originally to finance road infrastructures, rather than reduce congestion (Ramjerdi et al. 2004). Since 2003, however, only 45% of the revenues generated by the congestion charge of Bergen are still used to finance road constructions, while the rest is used to improve environmental quality and road safety. Users have to pay each time they enter the city center, but not when they exit. Users may also benefit from some discounts if they buy monthly, biannual or annual permits, which affect the marginal cost of commuting.

In 2003, London implemented a congestion charge of £5 per vehicle per day for either entering, or circulating within central London (Leape 2006). The rate has been revised upward several times and is currently at £15. As in other cities, exemptions include bicycles, motorcycles, taxis, people with disabilities, and buses. Residents pay only 10% of the charge when crossing or traveling within the London congestion charge zone. Revenues are used to fund public transportation (about 80%), road safety (11%), and cycling and walking projects (9%). The main objective of the London congestion charge was to reduce traffic and congestion. The drop in traffic between 2002 and 2003 has been estimated at about 30%, exceeding expectations. Transport for London estimated that most of the decrease was due to a switch to public transportation, and a small fraction related to the use of bicycles or taxis. However, about 25% of drivers had adapted their journey, avoiding the congestion charge, and up to 10% may be now traveling outside of charged areas. Average travel speeds also increased in central London, from 8.9 miles per hour to 10.4 miles per hour, based on a simple before-after comparison for 2003 (Leape 2006).

In Sweden, Stockholm implemented a congestion charge in 2007 after a trial period and a referendum, accepted by 53% of the city’s voters (Eliasson et al. 2009). Gothenburg followed in 2013, after a non-binding referendum, in which the congestion charge was rejected by 56% of voters, but nevertheless implemented. An important debate on the use of revenues, which were supposed to finance a rather unpopular rail tunnel under the city, might have contributed to its rejection (Börjesson and Kristoffersson 2015; Andersson and Nässén 2016). In both Gothenburg and Stockholm, road users have to pay to enter as well as to exit the city center. In contrast to other cities, taxis are not exempted in Stockholm, albeit they represent around 8% of the cordon passages. In Stockholm, following the implementation of the congestion charge, traffic volume decreased by about 20% and kilometers driven in the inner city by about 15% (Eliasson et al. 2009; Börjesson et al. 2012; Croci 2016). Travel times decreased between one third and one half during the peak periods and a 4.5% increase in the number of passengers by public transit is attributed to the road toll.

In 2008, Milan implemented a road pricing scheme, called Ecopass, to curb pollution. Ecopass was a cordon pricing scheme, charging a daily fee for entering the cordoned zone depending on the vehicle’s emissions of PM_10_ (Croci and Douvan 2015). Registered residents of the cordoned area received a 60% discount. In 2011, a majority of voters (79%) supported the extension of the road pricing scheme with a congestion charge called Area C. After a trial period, Area C became permanent in 2013. With the new scheme, highly polluting vehicles are outright banned from the city center, whereas all other vehicles pay a daily charge of €5. As its predecessor, Area C also includes a system of privileges for residents, who receive the right to cross the cordon 40 times a year for free, and then face a reduced charge of €2. Milan’s scheme also exempts alternative fuel vehicles, among others. Percoco (2013) provides an initial assessment of Area C, showing a decrease in charged vehicles of about 56% and an increase in the purchase of alternative fuel vehicles of about 5%. During a suspension of the scheme due to a court ruling, Gibson and Carnovale (2015) find that traffic in the cordoned area increased by up to 20%, while it decreased right before and right after Area C’s standard charging hours (by about 23%), and on the roads surrounding the cordoned area (by about 18%). While the charge was inactive, CO and PM_10_ emissions increased by 6% to 17% inside and outside Area C, respectively.

### Public Support

Public support is, in several contexts, the main obstacle to the implementation of environmental pricing. A rapidly growing literature has emerged to address this issue, and a few stylized facts have emerged (cf. Carattini et al. 2018b for a review).

First, public support tends to decline as the level of stringency, measured by the tax rate, increases. Choice-experiment surveys are particularly suited to detect this phenomenon (e.g. Sælen and Kallbekken 2011; Brännlund and Persson 2012; Gevrek and Uyduranoglu 2015; Carattini et al. 2017).

Second, people may have a tendency to overestimate the downsides of a policy change, and underestimate the upsides, in particular the incentive effect of environmental taxes and their ability to change behavior (e.g. Dresner et al. 2006; Steg et al. 2006; Baranzini and Carattini 2017; Carattini et al. 2017).

Third, it follows from the previous point that public support may increase after a policy is implemented and people experience it. This stylized fact follows from two types of studies. First, lab studies, in which trial periods are manipulated by the experimenters. Cherry et al. (2014) is the first study to exogenously allocate trial periods across experimental groups before a vote on a Pigouvian tax. Janusch et al. (2020) expand on this approach by looking specifically at a congestion game, in which players have heterogeneous time preferences and can vote on a congestion charge, before, during, or after a trial, which allows isolating the effect of learning via different trial durations on public support. Janusch et al. (2020) also randomize information provision, which in their context focuses on the costs of the congestion charge. Second, observational studies, comparing public support across control and treatment groups before and after the plausibly exogenous introduction of a Pigouvian tax. Carattini et al. (2018a) leverage the decision of the Supreme Court of Switzerland to impose the implementation of pricing garbage by the bag in a Swiss canton, part of which already uses this instrument. Pricing garbage by the bag substantially reduces residential waste with little unintended effects and this leads many voters to reconsider the policy. No change in perceptions is observed in the control group, which had already experienced the policy. This study confirms the findings of previous studies on congestion charges relying on simple before-after comparisons. In particular, Schuitema et al. (2010) and Hansla et al. (2017) study, respectively, the abovementioned referenda of Stockholm, and Gothenburg, which both followed a trial period, and show higher public support after the trial runs. Several other studies show that preferences and attitudes towards road pricing improve over time. For instance, after a single year of implementation, the fraction of surveyed people opposing the tolls decreased from 54% to 34% in Bergen, from 70% to 64% in Oslo, and from 72% to 48% in Trondheim (Odeck and Bråthen 2002). These findings are consistent with Winslott-Hiselius et al. (2009), who argue that a trial period contributes to making the benefits of a congestion charge salient to voters (see also Gu et al. 2018 for a review of the literature with specific focus on congestion charges).

Fourth, people tend to have a preference for earmarking the generated revenues for environmental purposes. Many people do not expect environmental taxes to change behavior, and thus do not expect any effect on the environment unless revenues are earmarked for environmental purposes. The review of road pricing schemes by Jaensirisak et al. (2005) suggests that ex-post acceptability is higher for schemes (19 in total) that earmark revenues for environmental purposes (an average of 55% of support) than for schemes (32 in total) in which revenues are not earmarked (35% of support). Earmarking guarantees that the tax is not implemented for fiscal purposes. Revenue redistribution, for instance by reducing existing, distortionary taxes, may also be seen with suspicion. People may not see the rationale for taxing here and redistributing there, especially when redistribution takes place in an area unrelated to environmental policy. Sælen and Kallbekken (2011) refer to this phenomenon as issue-linkage. In our context, since people may believe that drivers are inelastic to road charges (Ubbels and Verhoef 2006), they may then have a preference for earmarked revenues for public transportation. People may also like to vote on explicit policy packages, charging road transportation on one hand, and expanding public transportation in the other, as for instance in Stockholm (cf. Kottenhoff and Brundell Freij 2009).

Public support is most likely the main obstacle to the implementation of congestion charges (see again Gu et al. 2018 for a review). While in Sweden referenda on congestion charges were organized under the pressure of the central government, in several other contexts policymakers backed away at an earlier stage, as in Cambridge, Hong Kong (Ison and Rye 2005), and New York City. In New York City, people perceived a scheme charging traffic in Manhattan as particularly unfair, especially for people living in the outer boroughs. Also, proposed exemptions to taxi drivers, a significant contributor to New York City’s traffic, were a source of public opposition (Schaller 2010). New York City has more recently tried again to implement a congestion charge, with a slightly revised design compared to the earlier attempts. The charge was initially expected to be introduced in 2021 with revenues earmarked for public transit. However, the congestion charge is, at the time of writing, still pending approval from the federal government. In Manchester and Edinburgh, public ballots were organized and the congestion charges rejected by 79% and 75% of voters, respectively (Hensher and Li 2013). The double cordon scheme proposed in both cities was perceived as too complicated by the general public. In the case of Edinburgh, a survey shows that only 47.8% of the respondents knew the charge level that would have been implemented and only 37.4% believed that the scheme could reduce congestion (Allen et al. 2006). While in Gothenburg the referendum was non-binding, in Manchester and Edinburgh the project of a congestion charge was abandoned after the rejection on the ballot.

### The Geneva Congestion Charge

#### Rationale

About 75% of the Swiss population (8.4 million in 2016) lives in urban agglomerations, which cover about one quarter of the territory and provide 70% of the jobs (ARE 2018). Population density is thus relatively high, with most people being concentrated around major cities, connected by an intense network of roads and railways. If rail represented more than half of the passenger traffic in 1950 (40% for roads), in 2015 only 15% of the trips across Switzerland were undertaken by train (75% for roads, see Litra 2017). In absolute terms, road traffic volumes increased 15 times from 1950 to 2015, whereas train traffic 3 times only. Many city centers are located near the border with Germany, France, Italy, and Austria. About 2.2 million people cross the Swiss border every day, 96% of them by car (OFS 2017). As a result, the road network is often congested, especially around urban agglomerations. The direct cost of traffic jams are estimated to exceed one billion CHF a year (Keller and Wüthrich 2016), while the external costs from accidents and environmental pollution from driving at about CHF 8 billions a year (ARE 2016).^1^

According to the Swiss Constitution, public roads should be free of tolls. The road infrastructure is currently mainly funded by specific taxes, such as fuel tax and a highway sticker. The latter was introduced in 1994, after a public ballot. Since then, holding a vignette is compulsory for driving on highways. Ten years later, voters accepted an increase from CHF 30 to CHF 40 a year in the price of the vignette. A further increase to CHF 100 was, however, rejected in 2013 by 60% of the voters. Public ballots take place 3 to 4 times a year in Switzerland, with people voting in each occasion on about 3-4 subjects of national or local politics. While public support has been instrumental for the implementation of congestion charges in virtually all contexts where it exists, acceptance by voters is all the more necessary in Switzerland.

Until recently, mobility pricing in general, and congestion charges in particular, were only a theoretical concept in Switzerland. However, given the high level of traffic congestion, the Swiss government introduced mobility pricing to the policy agenda, and invited cantons and cities to step forward. Geneva was among the candidates for a congestion charge pilot program. According to the latest release of the TomTom Traffic Index, which ranks some 400 cities by their congestion levels using data from location devices, Geneva is a heavily congested city in which the average commuter loses about 146 hours per year due to traffic congestion, especially during weekdays (about 60% of extra time in the morning peak and about 70% of extra time in the evening peak). With more than 2 millions trips per day in the agglomeration of Geneva, of which more than half a million are undertaken in the city center, the cost of congestion is very large. A similar pattern emerges from INRIX’s Global Traffic Scorecard 2019, another ranking of almost 1,000 cities based on pollution levels.^2^

There are several reasons for this particular situation. First, similarly to many European cities, the city center was built before the advent of the automobile. Second, Geneva has a small territory with relatively high density, leading to a tight housing market (Drechsel and Funk 2017). As a result, the urban area is expanding into the neighboring areas of France, increasing the pressure on road infrastructures.

Geneva also suffers from an important air pollution problem. According to recent administrative data, the total external costs of PM_10_ and NO_x_ on health, life quality, buildings, forests, and agriculture reach almost CHF 120 millions a year (Baranzini et al. 2017). Furthermore, the concentration of most pollutants is higher in the city center than in the suburban areas. Concentrations of NO_x_ and PM_10_ per km^2^ are almost four times higher in the smaller perimeter than in the rest of the canton.

According to a recent survey on the quality of life in Geneva and surroundings, including the adjacent areas of France, 45% of respondents consider traffic the top policy priority in the region, up from 34% in 2016 (Baranzini et al. 2018b). It does not surprise, then, that the Canton of Geneva is willing to run a pilot scheme with a local congestion charge. Geneva tried several options to tackle congestion in the past, but many faced strong political resistance. Only recently, a project to build either a tunnel under, or a bridge over, the lake of Geneva to connect the right and left banks, which dates back to 1896, was accepted in a public ballot. In 2016, 68% of the population also accepted a policy package addressing mobility issues, including additional pedestrian areas, and bike lanes, as well as low emission zones. While this positive outcome is a strong signal that the local population is supportive of major changes in mobility, important concerns remain for policymakers on the potential acceptability of a congestion charge.

#### Design

**Fig. 1:**
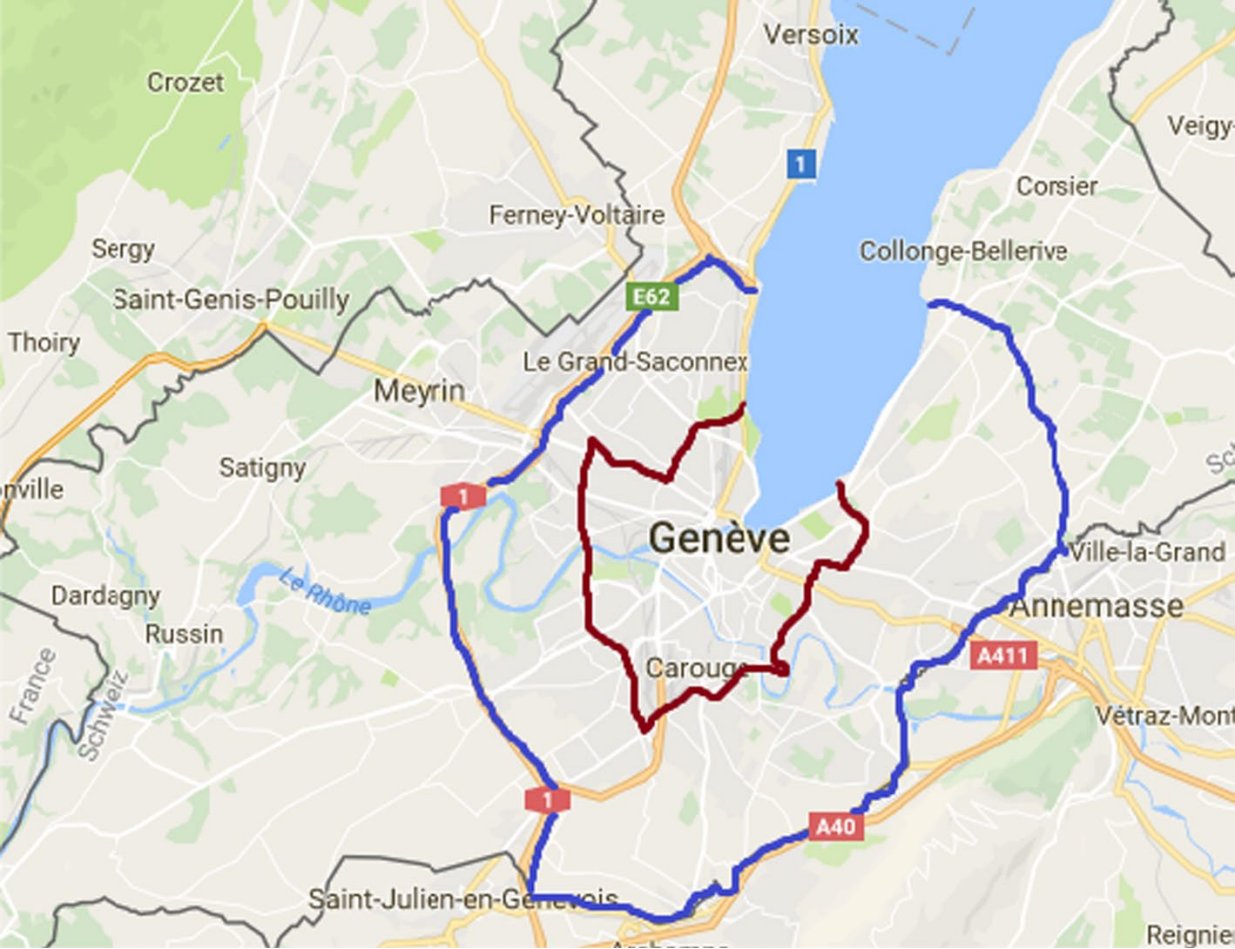
The two perimeters of the congestion charge: the highway ring (blue) and the urban center (red)

Following a decision by the local parliament, a task force was instituted to study different options for a potential congestion charge. This study is part of these efforts. In what follows, we consider a set of possible suitable designs for the context of Geneva. We test their acceptability by the general public in Geneva and the neighboring areas. Based on the local context, and lessons from existing schemes, we consider the following dimensions: charge rate, perimeter of the charge, modulation of the charge, level of exemptions, beneficiaries of exemptions, and use of revenues. Figure 1 illustrates the two options for the perimeter, which are given by the political process and the local topography. Being surrounded by France, it is legally difficult to conceive a perimeter either in France or right at the border. The perimeters that we consider include all the areas with very high levels of congestion in the region (“hotspots” identified by TomTom) and were validated by policymakers. First, a perimeter mainly overlapping the Geneva highway ring, which encircles most urban and suburban areas. Driving on the highway to bypass the city of Geneva would remain free of charge. Second, a perimeter around the urban center, where walking, cycling, and public transportation are already credible alternatives to driving, but motorized traffic remains important. Every day, more than 600,000 trips entering, leaving or crossing this area are undertaken. According to internal simulations by the local government, the number of trips to the urban center could be dramatically decreased with a congestion charge. For instance, implementing around the urban center a congestion charge set at CHF 1, with a CHF 1.50 top-up at peak hours and a 50% exemption to residents, could potentially lead to a reduction in traffic of about 50%. In either case, the congestion charge would use cordon pricing. With this design, users would be charged only when crossing the cordon, not for internal rides. We, however, also consider a distance-based charge in our survey.

In terms of pricing, we consider a CHF 0.20 - 5 range, to be paid, as in Stockholm, for both entering and leaving the zone in order to reduce both morning and evening congestion peaks. In Geneva, almost one fourth of the traffic takes place in the morning and evening peaks. We consider CHF 5 (CHF 10 for a two-way trip) as the upper bound of a politically plausible charge. We assume the congestion charge to be in force from Monday to Friday, with the exception of public holidays, from 6 am to 7.30 pm, the interval of time when congestion is the highest. For simplicity, we do not consider alternative timing options in this study.

However, we do consider five different modulation options, either on top or instead of the standard charge. First, we consider a top-up charge of CHF 1 during periods of high pollution, in response to variation in pollution levels (Coria et al. 2015), with the magnitude of the top-up charge being based on recent studies on the external cost of road traffic in terms of air pollution in Switzerland (Ecoplan/Infras 2018). Second, we consider a top-up charge of CHF 1.50 between 6.30 am and 9 am and between 4 pm and 7 pm, when congestion is at its peak (peak hours). The top-up charge is computed to reflect average time lost in traffic and the median wage in the local market. Third, we consider a top-up charge of CHF 0.20 for each kilometer driven inside the perimeter. With this option, we come close to the textbook ideal of pricing according to marginal damage, although, for ensuring comparability in the survey part of this study, we consider marginal pricing on top of the fixed charge of crossing the cordon. CHF 0.20 follows from the literature, which estimates the social cost of congestion in our and similar contexts (Maibach et al. 2007; Ecoplan/Infras 2014). Fourth, we consider a scheme in which the standard charge applies, but only during peak hours. During off-peak hours, crossing the cordon is free. Everything else equal, this modulation produces the lowest cost for drivers. Fifth, we consider a reference scenario wherein pricing is constant during the day, i.e. there is neither a top-up charge nor an uncharged period. For simplicity, we refrained from including real-time pricing, as implemented in Singapore, among the modulation options of this study.

In the existing road pricing schemes, subgroups of users are often totally or partially exempted. Some exemptions can be rationalized on efficiency grounds, others are implemented to increase perceived fairness and acceptability. In our study, exemptions can go from 0% (no exemption) to 100% (total exemption). Building on the existing schemes, we consider the following groups as potential beneficiaries of a partial or total exemption: people living within the cordoned area (residents); motorized two-wheel vehicles (e.g. motorbikes); professionals with an economic activity within the perimeter; electric vehicles; frequent commuters. Residents are exempted in most current schemes, essentially for fairness reasons. They have sometimes no alternative to crossing the cordon, for instance for special shopping or long-distance trips, and, as a result, cannot avoid the charge. They may, however, be its primary beneficiaries. In London, residents are exempted at 90%. In the case of Stockholm, Eliasson and Mattsson (2006) show that the congestion charge’s burden is borne mainly by residents of the cordoned area. Motorbikes are exempted in several cities, including Bergen, London, Milan, and Stockholm, mainly because they generate less congestion and pollution. Business rides can be exempted to limit adverse competitiveness effects on shops and businesses located within the area, beyond the potential variation in customers that the congestion charge may generate (e.g. Leape 2006). Electric vehicles do not generate less congestion, but they do generate less pollution, at least in the location where they are used. Highly polluted cities, such as London, exempt electric vehicles, possibly beyond the differential in marginal damage, to spur their adoption by private households and professional drivers. Frequent commuters are partially exempt in the Norwegian schemes and in Milan. While from an efficiency perspective each trip should be charged in the same way, from a fairness perspective there may be a rationale for reducing the marginal charge for people crossing the border particularly often. Following the Norwegian example, we define as frequent commuters people who would register for 200 journeys across the perimeter and would benefit from a reduced charge on the following 200.

Revenues generated from the congestion charge could be used in many ways. We consider five of them: earmarked for public transportation improvements; earmarked for measures improving road infrastructure; earmarked for measures tackling air and noise pollution; earmarked to fund a tunnel, or bridge, to cross the lake of Geneva; redistributed back to the population of the Canton of Geneva through a reduction in the annual vehicle registration fee. From an efficiency perspective, the best use of revenues would imply allocating them to the general budget for policymakers to determine their use based on the expected social return, which may include reducing distortionary taxes. However, we refrain to include the option to simply allocate the revenues to the general budget, as the literature points to this option as generally politically unfeasible. Reducing existing taxes, for instance on labor, tends also to be rather unpopular. Since there is a general consensus in the literature that using revenues in the same domain as the charge is generally better understood by the general public than an allocation to the general budget or a reduction in an non-related tax (Jaensirisak et al. 2005; Steg et al. 2006; Kallbekken and Aasen 2010), and given that reasons of power limit the number of possible attribute levels that we could test, the options that we consider imply either earmarking or rebating revenues in areas related to the congestion charge. That is, we follow the lessons from the literature on public support on the importance of issue-linkage. Further, in the context of Switzerland, the guidelines imposed by the Swiss Confederation to implement pilot programs for congestion charges explicitly excluded allocating revenues to the general budget.

Earmarking for public transportation improvements usually increases public support for road pricing schemes (Grisolía et al. 2015; Corvec et al. 2016), unless people do not expect additional funding for public transportation to lead to sizable improvements in their daily life (Allen et al. 2006). Public transportation improvements may not only benefit the residents of the Canton of Geneva, but also commuters from the surrounding areas. Similarly, improving transport infrastructure, including road and bicycle lanes, could generate benefits for individuals living in the areas surrounding the Canton of Geneva. According to the abovementioned 2016 ballot on mobility, a majority of citizens in the Canton of Geneva supported a policy allocating additional funding to mobility investments, including in favor of cyclists and pedestrians. In our context, earmarking for transport infrastructure is meant to cover part of the expenditures for those transport policy measures. Earmarking for air and noise pollution would address one of the main externalities of driving, beyond the incentive effect of the congestion charge. This measure would, however, mainly benefit the inhabitants of the Canton of Geneva, which would collect the revenues and spend them within its boundaries. The rationale for considering earmarking revenues to fund a road infrastructure to cross the Lake Geneva is twofold. First, the population of Geneva recently voted on a constitutional article supporting the construction of either a bridge or a tunnel. However, how exactly this infrastructure is going to be funded remains open to the decisions of lawmakers. Second, as discussed, the case of Gothenburg suggests that earmarking revenues for a specific, large investment may play a significant role in defining public support, especially if such investment is very controversial among the general public. Reducing the vehicle registration fee would meet the revenue-neutrality criterion requested by the federal government. It would imply a transfer among the vehicle owners of the Canton of Geneva from a one-off fee that does not depend on kilometers driven to a congestion charge. This redistribution scheme would, however, not benefit individuals whose vehicle is registered in another canton or country.

Table 2 summarizes the dimensions and levels considered for the design of the Geneva congestion charge, which correspond to the attribute and levels in the empirical analysis of public support.

**Table 2:** Congestion charge design: characteristics (attributes) and levels

Attributes						
	Perimeter	Charge rate	Modulation	Exemption	Beneficiaries	Revenues
Levels	Center	0.2	Constant	0%	Residents	Public transportation
	Ring	1	Peak hours only	25%	Motorbikes	Transport infrastructure
		2	Peak hours top-up	50%	Business deliveries	Pollution reduction
		3	Pollution top-up	75%	Electric vehicles	Tunnel or bridge
		4	Distance top-up	100%	Frequent commuters	Vehicle registration fee
		5				

## Methodology

### Survey Design

We analyze the question of acceptability as follows. We are interested in public support for different hypothetical policy designs. Hence, we opt for stated preferences and more precisely for a discrete choice experiment (DCE). A DCE allows evaluating individual choices and the relative importance of each characteristic (attribute level) of a given option (alternative). DCEs are deemed particularly suited to inform the choice and design of multidimensional policies (Hanley et al. 2001). The DCE follows from the random utility model (RUM). Most commonly the utility function is defined as additively separable:

$$\begin{aligned} U_{ij}=V_{ij}(X_{ij})+\varepsilon _{ij} \end{aligned}$$

where $U_{ij}$ is the unobservable utility that individual *i* associates with alternative *j*, $V_{ij}$ is the deterministic part of the utility that individual* i* associates with alternative *j* depending on its attributes (X), and $\varepsilon {}_{ij}$ is the error term, which represents a random component associated with individual *i* and alternative *j*. It follows that the probability that individual *i* chooses alternative *j* from the choice set $C_{i}$ equals the probability that the utility associated with alternative *j* exceeds that associated with all other alternatives *h* of the choice set. This implies:

$$\begin{aligned} P(j|C_{i})=Pr[(V_{ij}(X_{ij}) - V_{ih}(X_{ih}) ) > (\varepsilon _{ih} - \varepsilon {}_{ij}){]}\, \forall  {h} \hbox { in } C_{i} \hbox { and } h\ne j \end{aligned}$$

We usually assume that the error terms are independently and identically distributed with an extreme-value distribution. This implies that the probability of alternative *j* being chosen over all other alternatives in the choice set can be expressed as following a logistic distribution (McFadden 1973). The conditional logit model follows:

$$\begin{aligned} P(j|C_{i})= \frac{exp(\mu V_{ij})}{\underset{h}{\sum }exp(\mu V_{ih})} \end{aligned}$$

where $\mu$ is a scale parameter.

This model is estimated by maximum likelihood.

In our DCE, each respondent is requested to answer to 10 different choice tasks. Each choice task includes three alternatives: two different congestion charge designs, leveraging the simplicity of pairwise comparison, and the status quo. The rationale for including the status quo is threefold. First, this survey is designed to replicate as closely as possible the context of a public ballot, thus mimicking an actual choice context (Harrison 2006). In a public ballot, voters have generally the possibility to refuse all options and maintain the status quo. Second, the status quo ensures that respondents are not forced to choose an alternative they may not desire (Hanley et al. 2001). If the status quo is preferred to the proposed alternatives, a model that does not include the option of rejecting all alternatives would lead to inaccurate estimates, as respondents would be forced to choose an alternative that provides a lower utility than the status quo. Third, the status quo provides us with the possibility to measure the overall acceptability of a congestion charge in Geneva, and not only the relative preference for a given attribute level.

Each congestion charge design in our survey results from the combination of the different levels of the six attributes presented in Table 2: charge rate, perimeter of the charge, modulation, level of exemption, exemption beneficiaries (if any), and use of revenues. When designing the DCE, we consider both statistical efficiency, which implies minimizing the length of confidence intervals, as well as response efficiency, which implies minimizing potential measurement error due to respondent inattention (Reed Johnson et al. 2013). A perfectly efficient design is balanced and orthogonal, which means that each level, and pair of levels, appear an equal number of times within an attribute, and the design, respectively. Our design relaxes the restriction of minimal overlap to allow a modest degree of level overlap. This means that the same level of an attribute can appear twice in the same choice task, but the very same combination of attributes and levels (duplicates) cannot. Respondents use heuristics to simplify decisions. They may for example eliminate an alternative if an attribute has a specific level, without even considering the other attribute levels. Level overlap can thus improve the precision of the utility estimates.

To improve the measurement of the coefficients of interest, each respondent receives one of 100 randomly-generated, pre-tested, versions of the questionnaire.^3^ To avoid order effects, the order of choice tasks is randomized within versions.

The survey was structured as follows. Prior to voting on the 10 hypothetical ballots, each respondent received general information about the local context, including figures on the level of traffic in Geneva, and the functioning of a congestion charge. We explained to respondents that the implementation of such a mechanism in Geneva would allow a better use of road infrastructures, reducing traffic at rush hours, and air pollution. In our introductory text, we stressed that the impact on traffic, the environment, the household’s purchasing power, and the generated revenue would depend on the specific design of the congestion charge that will be implemented (if any). After these introductory explanations, we provided a description of the attributes and levels. At each point in time during the completion of the choice-experiment part of the survey, respondents could access essential information, describing each attribute, through tooltips. This information is reported in Table 8. In providing this information, we reproduced the structure of an official ballot. Indeed, in Switzerland, weeks before the ballot day, each voter receives by post a set of voting materials, which includes detailed information on the items in the agenda. The survey instrument can be found in the Appendices.

An important, and original feature of this survey is that, on top of the discrete choice experiment, we use a split sample design to test the impact on public support of randomized information provision concerning the effectiveness of existing congestion charge schemes. We use two separate treatments. Hence, about two thirds of our sample receive either one or the other treatment, while the remaining third represents our control group, which is subject to the baseline level of information provided to all respondents. This approach allows us to causally infer on the effect of informational treatments on public support. Our treatments focus on two potential benefits of congestion charges, respectively. The first treatment stresses the benefits of congestion charges in reducing congestion, drawing from the experiences of existing schemes for which empirical evidence on traffic is available (Leape 2006; Eliasson et al. 2009; Percoco 2013). The second treatment stresses the benefits of congestion charges in reducing pollution, and noise, drawing from the experiences of existing schemes for which empirical evidence on air pollution is available (Croci 2016). The exact wording of each treatment, as translated from French, is provided in Table 3.

**Table 3:** Randomized informational treatments

Group	Information given
Congestion	We would like to remind you that the goal of the congestion charge is to reduce congestion. In London and Milan, congestion decreased by 30% and 25%, respectively, following the implementation of a congestion charge. In Stockholm, traffic was reduced by more than 20%. We expect similar effects in Geneva.
Pollution	We would like to remind you that the goal of the congestion charge is to reduce pollution and noise due to traffic. In London and Stockholm, small particles decreased by 10 to 15% and carbon dioxyde by 13 to 16% following the implementation of a congestion charge. The decline in pollution has had a positive impact on public health. In addition to improvements in air quality, the level of noise declined as well. We expect similar effects in Geneva.
Control	Only standard information provided

The rationale for these randomized treatments is the following. While the literature on public support emphasizes the information asymmetries between experts, in particular economists, and the general public, with lay people overestimating (underestimating), especially ex ante, the drawbacks (the benefits) of new environmental taxes, the causal effect of informational campaigns, addressing these information asymmetries, remains largely unexplored. In their review of voting behavior on road pricing, Hensher and Li (2013) emphasize the importance of information deficits as one of the main barriers to public support. People’s understanding of the effects of a congestion charge, and perception of its effectiveness, strongly correlates with public support. According to Eliasson and Jonsson (2011), beliefs on the potential effects of the congestion charge played a crucial role in its approval in Stockholm following a trial period. Local policymakers, in particular, emphasized the potential benefits from the congestion charge in terms of better air quality (which eventually materialized, as examined in Simeonova et al. 2018).

Hence, additional information needs to be provided to voters before a ballot to ensure that they take an informed decision. There is, hence, a strong rationale for trial periods. However, trial periods require, themselves, sufficient political buy-in. An alternative is represented by information provision through informational campaigns. In this respect, Carattini et al. (2017) and Carattini et al. (2019) analyze public support for carbon taxes by providing information on their impacts assessed with general computable equilibrium (CGE) models. Carattini et al. (2017) analyze voting behavior on an energy tax initiative, rejected by the Swiss population, and compare it through a discrete choice experiment with alternative policy designs, whose effects on different outcomes are provided to respondents as simulated by a CGE model. Carattini et al. (2019) provide information from a CGE model of the world economy to survey respondents in Australia, India, South Africa, the United Kingdom, and the United States to analyze public support for a global carbon tax or a global system of harmonized carbon taxes. In this paper, we push the frontier further by testing directly the provision of additional specific information to randomly-selected subsamples for causal inference in combination with a discrete choice experiment.^4^

### Data and Descriptive Statistics

The survey was administered online in September 2017 by a professional market research company with the goal of obtaining about 1,500 responses. We recruited individuals of adult age (at least 18 years old) living in the Canton of Geneva, the surrounding regions of Switzerland (the district of Nyon in the Canton of Vaud) and of France (Annemasse, Bas-Chablais, Genevois, and Pays de Gex). Respondents were informed that the study was conducted in partnership with the local authorities and that their response could impact actual policymaking. Such an approach builds on Harrison and List (2004) and was already applied in Switzerland by Carattini et al. (2017). Respondents did not receive any monetary compensation. The survey was completed by 1,430 respondents, which corresponds to 90% of those who acknowledged receipt of our invitation to fill the survey, but a smaller fraction of all prospective respondents who were contacted by the market research company. The final sample, composed of valid questionnaires only, covers 1,414 respondents. In this section, we compare our sample with the underlying population and comment on its representativity.

Table 9 in the Appendices displays the summary statistics for the socioeconomic variables collected in our survey. Swiss residents are overrepresented by design, since our focus is mainly on the political constituency that could affect the outcome of a potential ballot on the Geneva congestion charge. Hence, Table 11 compares our Geneva-based sample with the characteristics of the underlying population of the Canton of Geneva. In Table 12 we compare our entire sample to the entire region, known as “Grand-Genève”. In either case, if anything, we slightly overestimate the number of cars per inhabitants and the fraction of low-educated people, which may lead us to underestimate public support. Finally, Table 10 in the Appendices provides the standard balance of covariates. Table 10 shows that the three groups to which respondents were randomly assigned (pollution, congestion, and control) are very well, albeit not perfectly, balanced in terms of covariates. As per standard procedure, we thus include covariates as control variables in our empirical estimations of the treatment effects.

## Empirical Results

### Attributes

In this section, we discuss the overall level of public support and the relative preference for each attribute. We begin by presenting a set of descriptive statistics for our main outcome variables, in Table 4. Overall, 23% of the respondents reject all proposals of a congestion charge, no matter the design. Inversely, 23% of our sample always accepts one of the two congestion charge schemes proposed in each hypothetical ballot. For the remaining 54%, public support is contingent on the design. The average level of public support, measured as the number of accepted schemes over the total number of votes, is relatively high in our sample, at about 53.66%. Note that two thirds of our sample are subject to additional information. For one specific design, average public support reaches 65%. This design implies a small perimeter, at the boundaries of the city center, a price of CHF 0.2 per trip applied only during peak hours and only to non-residents (residents are fully exempted), and revenues earmarked for investments in public transportation. While the design that receives most support is relatively unambitious, there are other designs, which we may expect to have a stronger effect on commuters’ behavior, that receive majority support. In what follows, we identify the attributes and characteristics that increase, or decrease, public support. We consider that the goal for policymakers is not to find a design that creates unanimity, but get legislation passed.

**Table 4:** Public support: summary statistics for our outcome variables from the DCE

	Overall public support (%)	Accept all (%)	Reject all (%)	Design dependent (%)
Entire sample	53.66	23.34	22.50	54.16
*Residence location*				
Living within the perimeter	57.77	28.85	20.83	50.32
Living in the Canton of Geneva, but outside the perimeter	51.28	23.26	25.38	51.16
Living outside the Canton of Geneva	50.35	18.04	23.27	58.65
*Commuting mode*				
Commuting by car	48.90	17.20	25.17	57.63
Commuting by motorbike	50.85	27.85	30.38	41.77
Commuting by car or motorbike	49.19	18.75	25.93	55.32
Commuting by public transportation, cycling and walking	59.68	27.09	16.52	56.39
*Commuting frequency*				
Living in the Canton of Geneva	55.79	27.38	21.95	50.67
6-7 trips/week to Geneva	53.31	22.29	24.54	53.17
1-5 trips/week to Geneva	51.98	20.34	21.88	57.78

Public support seems to vary also along standard voter characteristics. On average, public support is higher among residents than for the remaining respondents, and among individuals who already commute by public transport, cycling, or walking. In what follows, we also analyze the role of heterogeneity across voters.

We now analyze the main set of findings concerning people’s preferences for the different attributes, and levels, covered by the survey. To this end, we bundle all observations together. We note that our analyses do not find any evidence of fatigue and learning effects and no significant variation on the time spent per task across tasks.

Table 5 provides the main results. Table 5 displays average marginal effects, for each attribute level, as estimated by the conditional logit model. Column (1) provides estimates for the full sample. Column (2) restricts the sample to the residents of the Canton of Geneva and column (3) to respondents living outside the Canton of Geneva. Only members of the second group would be entitled to vote, in a cantonal ballot in Geneva, on the congestion charge. Note that, as shown in Fig. 1, the perimeter covers only part of the Canton, even when located on the highway ring. Hence, a potential ballot would include as voters both people from the urban areas within or close to the perimeters and people from the adjacent suburbs and countryside. This makes the situation of Geneva relatively similar to that of Stockholm. Recall that the referendum on the congestion charge was held in the city of Stockholm, as well as in several neighboring municipalities experiencing different degrees of congestion. This contrasts, for instance, with Edinburgh, where only residents of the city were entitled to vote.

**Table 5:** Estimates from conditional logit: full sample, voters, and non-voters

	(1)		(2)		(3)	
	Full sample		Potential voters		Non-voters	
*Charge rate*						
CHF 0 (reference)						
CHF 0.2	− 0.051***	(0.017)	− 0.065***	(0.023)	− 0.034	(0.025)
CHF 1	− 0.109***	(0.017)	− 0.099***	(0.024)	− 0.119***	(0.025)
CHF 2	− 0.195***	(0.017)	− 0.173***	(0.023)	− 0.22***	(0.024)
CHF 3	− 0.262***	(0.017)	− 0.232***	(0.023)	− 0.296***	(0.025)
CHF 4	− 0.283***	(0.017)	− 0.239***	(0.023)	− 0.34***	(0.024)
CHF 5	− 0.33***	(0.017)	− 0.291***	(0.023)	− 0.375***	(0.025)
*Perimeter*						
Center (reference)						
Ring	− 0.008	(0.007)	0.0001	(0.009)	− 0.02*	(0.011)
*Modulation*						
Constant (reference)						
Peak hours only	0.018**	(0.009)	0.009	(0.012)	0.029**	(0.014)
Peak hours top-up	0.008	(0.009)	− 0.0003	(0.012)	0.017	(0.014)
Distance top-up	− 0.045***	(0.01)	− 0.073***	(0.013)	− 0.009	(0.015)
Pollution top-up	− 0.008	(0.009)	− 0.016	(0.012)	0.001	(0.014)
*Exemption level*						
0% (reference)						
25%	0.002	(0.009)	0.0004	(0.013)	0.004	(0.014)
50%	0.036***	(0.009)	0.036***	(0.013)	0.036**	(0.014)
75%	0.044***	(0.01)	0.041***	(0.013)	0.049***	(0.014)
100%	0.069***	(0.01)	0.046***	(0.013)	0.098***	(0.015)
*Beneficiaries*						
Business deliveries (reference)						
Residents	0.029***	(0.01)	0.060***	(0.013)	− 0.014	(0.014)
Motorbikes	− 0.015	(0.010)	− 0.001	(0.014)	− 0.035**	(0.015)
Frequent commuters	0.016*	(0.01)	0.022*	(0.013)	0.006	(0.014)
Electric vehicles	− 0.023**	(0.010)	− 0.003	(0.013)	− 0.05***	(0.016)
*Use of revenue*						
Vehicle registration fee (reference)						
Public transportation	0.082***	(0.010)	0.084***	(0.013)	0.080***	(0.015)
Transport infrastructure	0.050***	(0.01)	0.056***	(0.013)	0.044***	(0.015)
Pollution reduction	0.051***	(0.010)	0.062***	(0.013)	0.039**	(0.016)
Tunnel or bridge	0.035***	(0.010)	0.025*	(0.014)	0.05***	(0.016)
Number of respondents	1414		782		632	
Number of observations	42,408		23,454		18,954	
Pseudo $R^{2}$	0.0748		0.0523		0.1148	

Estimates report average marginal effects from conditional logit

Heteroscedasticity-robust standard errors in parentheses

Continuous *p*-values are provided in Table 21

*$$p<0.1$$, **$$p<0.05$$, ***$$p<0.01$$

**Fig. 2:**
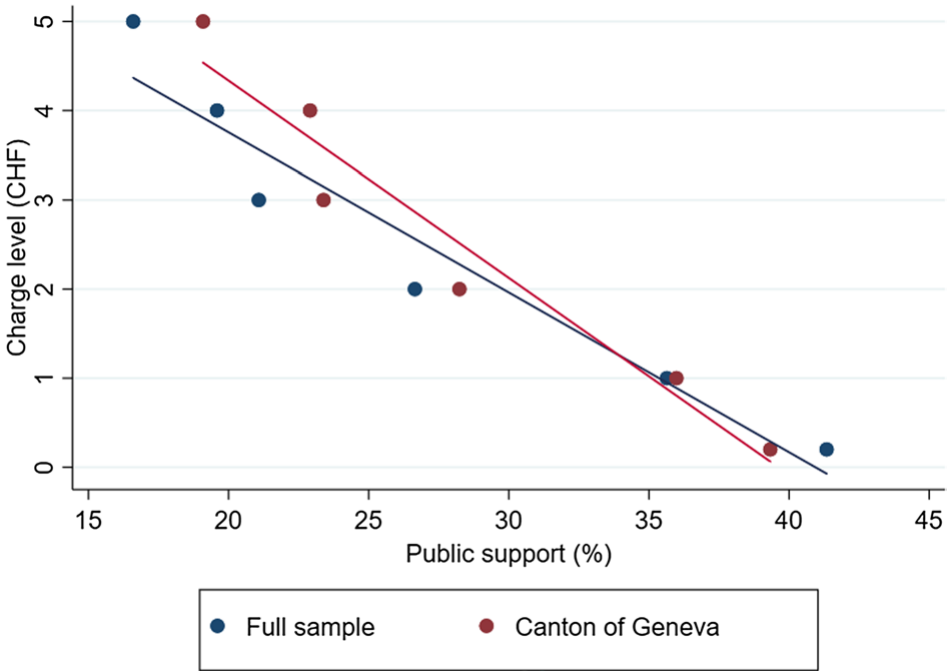
Charge rate and public support

A standard public choice result is that, the higher the level of a proposed charge, the lower its acceptability. This result is confirmed in Table 5, where public support decreases almost linearly with the charge. Figure 2 illustrates for each charge rate the average public support, across attributes and levels, for both the full sample and the subsample of potential voters. Even with very low charge rates, public support never exceeds 50% when the average over all attributes and levels is taken. As mentioned, however, public support can reach majority for some specific designs.

Figure 2 suggests that people in the Canton of Geneva tend to be significantly less sensitive to higher charge rates than the remaining individuals in the sample. Note that people living in the Canton experience both the direct benefits of the congestion charge, through a reduction in congestion and pollution, and the indirect benefits from the use of revenues, which takes place mainly at the local level. Table 13 in the Appendices reproduces the analysis of Table 5 splitting our sample into three groups: people living within the perimeter and people living outside the perimeter, either in the Canton of Geneva or in the surrounding areas. Note that since the perimeter changes with the design, the answers of some people are attributed to one group for some choice tasks, and to the other group for others. As shown in Table 13, non-residents, especially those not living in the Canton of Geneva, tend to be much more sensitive to the charge rate than residents. The divergence starts, in statistical terms, at a charge rate of CHF 2. In what follows, we consider people’s preferences for the other attributes, analyzing the behavior of the full sample, based on Table 5, as well as comparing residents and non-residents, based on Table 13.

When taking the entire sample, we observe no specific preference for either perimeter, i.e. a smaller perimeter corresponding to the urban center, and a larger one, located on the highway ring. This may be due, however, to the fact that the sample as a whole includes both people who would benefit from a smaller perimeter, as well as people for which the prediction is more ambiguous. Non-residents are expected to have a preference for a smaller perimeter, since they do not experience the benefits of a congestion charge, except perhaps for a faster commute, and with a smaller perimeter they would save money on all trips with a destination point between the two perimeters. For those who switch from one subsample to the other, the effect is ambiguous. Depending on where they work, with a larger perimeter they may no longer need to cross the cordon to commute. Note that about 57% of them work in the area within the smallest perimeter. Furthermore, with a larger perimeter, they may have the chance to live within the area and enjoy a better living environment, and to be eligible for potential exemptions for residents when crossing the cordon.

In Table 13, we do find a difference when comparing residents and non-residents. Non-residents living outside the Canton of Geneva have, as expected, a preference for a smaller perimeter. Currently, 26% of them work between the two perimeters and 88% commute by car or motorbike. Another 40% of our respondents live in the Canton of Geneva but outside the large perimeter. 40% of them work in-between the two perimeters and 60% commute by car or motorbike.

We now turn to the preferences for various modulation options. We observe, in general, that the least preferred option is the one involving a surcharge based on kilometers driven. No preference is observed for a top-up, be it based on pollution or peak hours, compared to a constant pricing. This confirms the trade-off, observed in the literature, between efficiency and public support. As suggested by Li and Hensher (2010) and Hensher et al. (2013), people may have a preference for fixed over variable charges, because their effect on the household budget is more predictable. In the survey, we only consider top-ups, i.e. additions that make the charge higher. In the specific case of the distance top-up, however, we should note that privacy concerns may also influence public support (or lack thereof). While this modulation integrates more closely marginal damages into marginal costs, the associated system could also allow the government to track with relatively high precision the movements of each citizen. In Hong Kong, for instance, the implementation of such a system was opposed on privacy grounds (Hau 1990; Khan 2001). According to Table 5, the most popular modulation implies a charging scheme that only applies to peak hours, which is the least stringent option, everything else equal. This option, as shown in column (3) of Table 5 and in Table 13, is favored in particular by people living outside the Canton, who may be able to adjust the timing of part of their rides through the congestion area. Note that 64% of them work in either one of the perimeters and a large majority of them commute by car or motorbike. Table 14 confirms that, in general, people commuting by car or motorbike would have a preference for a congestion charge that only covers peak hours.

From Table 5, we observe that, everything else equal, a higher exemption level leads to higher public support. This result is consistent with the effect of the charge rate. The relationship between the level of exemption and public support is again relatively linear. The same effect is observed for both the full sample and the subsample of potential voters. Note, however, that inhabitants of the Canton of Geneva, as shown in column (2), tend to be slightly less generous in the provision of exemptions. This makes sense. In most designs, revenues are recycled in a way that favors only, or mostly, people living in the Canton. Moreover, these would also benefit more from less congestion and better environmental quality.

In terms of beneficiaries, we find, as expected, a strong support for (partial) exemptions for residents living within the cordon. Unsurprisingly, this result is driven mainly by people living within the cordon, as shown in Table 13. That is, a trade-off between efficiency and acceptability may also be present when it comes to exemptions. As discussed, partial exemptions may be efficient if some vehicles are likely to generate less externalities. However, in our sample exemptions to motorbikes and electric vehicles receive, if anything, lower public support than business deliveries, the reference category. According to the Federal Office of Statistics, the penetration of electric vehicles in Switzerland is still very low, at about 0.3% of the total fleet in 2017. In the Canton of Geneva, in 2017, there were only 530 electric cars and 150 electric scooters, which also corresponds to the 0.2-0.3% range. The only group that seems to strongly support exemptions for motorbikes, are the bikers themselves, as shown in Table 16. A strong preference, driven by the response of residents, is found for exemptions to frequent commuters. Although efficiency dictates that each ride should be charged in the same way, everything else equal, fairness reasons could dominate people’s preferences. To further analyze the preference for exemptions for frequent commuters, we divide the sample into two subsamples, based on the number of journeys per week to Geneva. Table 15 presents the relevant findings. As shown in column (2), the preference for partial exemptions for frequent commuters is mainly driven by people currently driving across the hypothetical cordon about 6-7 times a week.

In terms of revenue recycling, in line with the literature, we observe a strong preference for earmarking for improvements in public transportation. The preference for public transportation over the other revenue use options is shared by all subgroups analyzed in Tables 13, 15, and 16. The revenue-neutral option of redistributing revenues back to the population through a reduction in the vehicle registration fee, the variable of reference, is the least popular option for all groups, including the inhabitants of the Canton of Geneva, who would benefit from it. This finding is also in line with the literature. People may tend to have a preference for earmarking over revenue-neutral designs, even when there is a direct linkage between the new charge and the mode of rebate. We also find little support for financing a tunnel or bridge across the Lake Geneva. This result mirrors the case of Gothenburg, where part of the opposition to the congestion charge was related to the use of its revenues for a rail tunnel under the city (Börjesson and Kristoffersson 2015; Andersson and Nässén 2016).

Overall, we find that most design parameters affect public support. Within designs, differences across respondents seem to be mainly due to the respondent’s residence location and commuting mode. That is, the standard public choice tenet that people’s preferences are mainly driven by their own interests seem to be largely confirmed in our context (see Downs 1957; Kramer 1983; Ferejohn 1986). However, no matter the different individual characteristics, we confirm the important trade-off between efficiency and acceptability. Section 4.3 further investigates the role of heterogeneity within our sample.

### Information

**Fig. 3:**
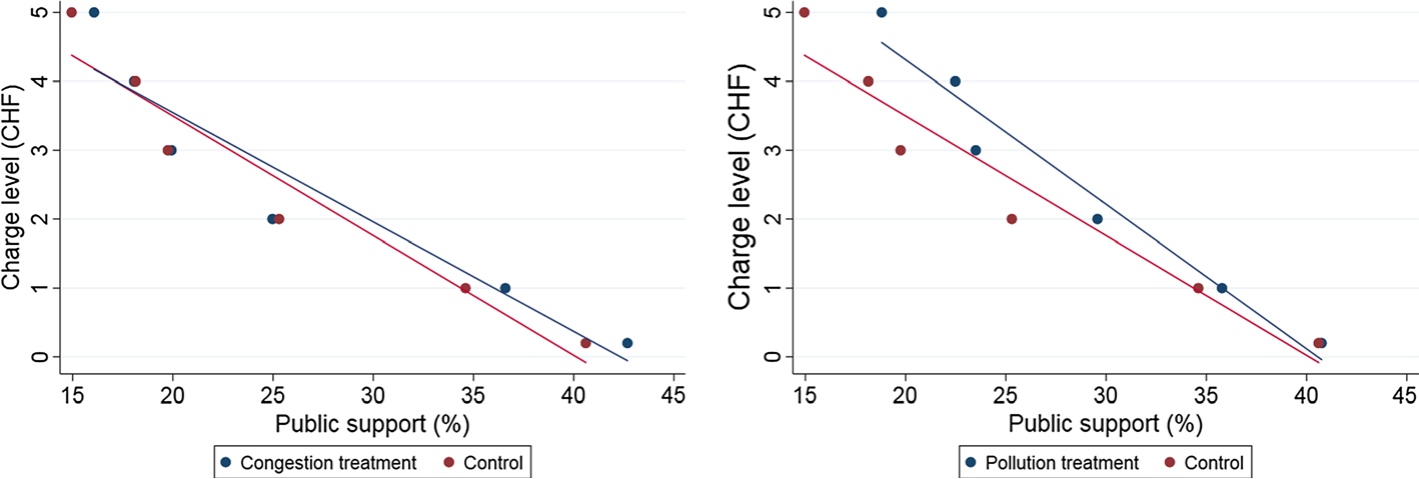
Informational treatments, charge rate, and public support

In this section, we analyze the impact of the randomized informational treatments on public support for the different designs. Overall public support, measured again as the number of votes in favor of a congestion charge over the total number of votes, amounts to 51.07% in the control group, 52.83% in the Congestion treatment, and 57.07% in the Pollution treatment. Figure 3 compares public support across different charge rates for each treatment compared to the control group. The left panel shows the Congestion treatment, the right panel the Pollution treatment. Both treatments tend to increase public support, but the Congestion treatment does so only marginally. Stressing the observed effects of existing congestion charges on traffic may not affect behavior in our sample. People in our sample may tend, in general, to factor in the effect of a congestion charge in reducing traffic. Note that in French, and so in our survey, the term for congestion charge is “péage urbain” (urban toll), which does not explicitly relate to congestion.

The difference in public support in the control group and in the Pollution treatment is very small at low charge rates, when public support is relatively high, but increases with policy stringency. To test whether this difference is statistically significant and to assess the pattern of divergence, we analyze the causal effect of both randomized treatments on public support at each charge rate. Table 6 displays the results (see Table 17 in the "Appendix" for the inclusion of control variables, to which our results are robust). As expected following Fig. 3, regardless of the charge rate, the Congestion treatment has no significant impact on public support. The difference observed in Fig. 3 is not only marginal, but also statistically insignificant. The coefficient is also statistically insignificant for the Pollution treatment, as long as the charge rate remains below CHF 2. Starting from CHF 2, we observe a statistically significant divergence in public support between the Pollution treatment and the control group.

**Table 6:** The impact of informational treatments on public support, for each charge rate

	(1)	(2)	(3)	(4)	(5)	(6)
	CHF 0.2	CHF 1	CHF 2	CHF 3	CHF 4	CHF 5
Congestion	0.020	0.020	− 0.003	0.002	− 0.001	0.012
	(0.018)	(0.017)	(0.016)	(0.015)	(0.015)	(0.014)
Pollution	0.002	0.012	0.042**	0.037**	0.042***	0.038***
	(0.017)	(0.017)	(0.016)	(0.014)	(0.014)	(0.013)
Number of respondents	1414	1414	1414	1414	1414	1414
Number of observations	4,736	4,711	4,702	4,702	4,711	4,710
$R^{2}$	0.0003	0.0002	0.0019	0.0017	0.0027	0.0021

Estimates report average marginal effects from logit

Heteroskedasticity-robust standard errors in parentheses

Continuous *p*-values are provided in Table 22

**$$p<0.05$$, *** $$p<0.01$$

In Table 7, we test whether this result carries over also to different modulations (see Table 18 in the "Appendix" for the inclusion of control variables, to which our results are robust). Recall that we consider several options that deviate from constant pricing throughout the day, including a series of top-up charges. We are now interested in analyzing the impact of the informational treatments on public support for each modulation, i.e. whether the randomized informational treatments also lead to higher support for more stringent designs. While the effect of the Congestion treatment remains marginal, we do observe higher support for a peak hour top-up as well as for a pollution top-up. Support for the pollution top-up increases by about 5%. Hence, the findings show that, in our survey, the randomized informational treatments contribute to close the gap between efficiency and acceptability.

**Table 7:** The impact of informational treatments on public support, for each modulation

	(1)	(2)	(3)	(4)	(5)
	Constant	Peak hours only	Peak hours top-up	Distance top-up	Pollution top-up
Congestion	0.019	− 0.007	0.001	0.025	0.006
	(0.015)	(0.015)	(0.015)	(0.014)	(0.015)
Pollution	0.018	0.017	0.046***	0.022	0.045***
	(0.014)	(0.015)	(0.014)	(0.014)	(0.014)
Number of respondents	1414	1414	1414	1414	1414
Number of observations	5’638	5’678	5’646	5’659	5’651
$R^{2}$	0.0003	0.0004	0.002	0.0007	0.0018

Estimates report average marginal effects from logit

Heteroskedasticity-robust standard errors in parentheses

Continuous *p*-values are provided in Table 23

***$$p<0.01$$

### Heterogeneity

In this section, we further investigate the role of heterogeneity across individuals to better understand how preferences vary with voter characteristics. To this end, we apply a latent-class model. We can identify 5 latent classes in our data.^5^ The number of respondents per class goes from 201 (class 5) to 393 (class 1). Overall public support varies between 97% (in Class 1) to 1.37% (in Class 4). Hence, we can define Class 1 as (virtually) always in favor, and Class 4 as (virtually) always against. Overall public support is 60%, 36%, and 76% in Classes 2, 3, and 5, respectively.

Table 19 in the Appendices shows how attribute and level preferences change across classes, based on a conditional logit model. In Table 20, also in the Appendices, we use a multinomial logit model to analyze the composition of the different classes.

This analysis provides a set of additional findings. While over the whole sample we observe a clear negative relationship between the level of the charge and public support, the latent class analysis points to varying degrees of price sensitivity as well as a preference, in three classes, for a moderate charge over no charge. In general, Classes 1 and 2 tend to be relatively price inelastic, while in Classes 3, 4, and 5, public support reacts much more strongly to an increase in the charge. In Class 1 and 5, there is a preference for a positive charge compared to no charge at all, after which the standard negative relationship between charge rate and public support is observed. The other differences among Classes 1, 2, and 5, which all show (relatively) high support, relate with modulation and use of revenue. In Class 2, no modulation is statistically preferred to a constant modulation. Class 2 also shows a preference for large exemptions, especially for residents, frequent commuters, and electric vehicles. Class 3 shows the strongest price sensitivity. While a small charge is preferred to the status quo, public support drops rapidly as the charge increases. Respondents in Class 3 also exhibit strong preferences for large exemptions, similarly to Class 5. Respondents in Class 5 tend to favor revenue earmarking for the tunnel, or bridge, crossing the Lake Geneva.

Members of Class 1 are more likely to be residents of the Swiss Cantons of Geneva and Vaud and located within the area of the hypothetical perimeters. Members of Class 2, and Class 3, tend to be relatively younger than the rest of the sample. Car and motorbike commuters are overrepresented in Class 3. Members of Class 4 tend to be residents of the Swiss Cantons of Geneva and Vaud, who commute by car or motorbike. Members of Class 5 are more likely to live in France, hence the preference for the tunnel, or bridge, crossing the Lake Geneva, which would benefit mainly individuals living in the surrounding of Geneva and trying to bypass part of the city center.

## Conclusions

Economists have long advocated for congestion charges to internalize the externalities of driving. However, only a few cities in the world have implemented a congestion charge. Others have considered it, but later abandoned it before lawmakers would suffer a political defeat. In some other cities, proposals for a congestion charge were abandoned after an unsuccessful public ballot.

Switzerland recently changed its regulation to allow congestion charge trials in a number of cities willing to act as forerunners. Geneva, one of the most congested cities in the world, stepped forward. Policymakers are currently considering a potential design to be trialed over the next few years. In a country where people vote very often, reaching sufficient public support for such a radical change in transportation policy represents a sine qua non. This paper builds on the theoretical literature, and draws lessons from the existing congestion charge schemes in the world, to put forward a set of plausible designs for a Geneva congestion charge. Then, it evaluates public support for each of them, using a large survey of respondents from Geneva and the surrounding regions of both Switzerland and France. The literature on public support for environmental policy suggests that acceptability may change dramatically with the design. Hence, public support for each policy design is tested with a discrete choice experiment. The parameters considered for the design are the following: charge rate, perimeter of the charge, modulation of the charge, level of exemptions, beneficiaries of exemptions (if any), and use of revenues. According to the literature, information asymmetries may represent another obstacle to public support. The general public may not expect congestion charges to work as well as economists do. However, no causal evidence on the effect of additional information on public support for congestion charges has so far been provided. With a split sample design on top of the standard discrete choice experiment, we test the effectiveness of two randomized informational treatments stressing the benefits of congestion charges for abating pollution and reducing congestion, respectively.

Our findings confirm the importance of design for public support. Public support decreases (increases) considerably when increasing the level of the charge (exemptions), although important heterogeneity in the sample is observed and some groups tend to be much more sensitive to the level of the charge than others. Heterogeneity determines most of the findings in our paper. Preferences for either a more compact perimeter or an extended area depend on where people live and how they commute. Our findings also highlight an important trade-off between acceptability and efficiency. While on efficiency ground congestion charging should match as closely as possible the marginal damage of driving, people tend to have a strong preference for a constant, predictable modulation. Similarly, people do not favor exemptions to the vehicles causing less congestion or pollution, such as motorbikes, or electric vehicles. Only bikers support exemptions for motorbikes. Residents demand exemptions for residents. Frequent commuters have a preference for a scheme providing a discount when prepaying for many rides, as in use in Norwegian cities. Most people demand earmarking for improvements in public transportation. Revenue-neutral schemes, favored by the Swiss federal authorities, are especially disliked by the general public. Finally, we show that information asymmetries do contribute to lower public support, as our randomized informational campaigns contribute to higher public support. That is, in the context of our study, we find that tackling information asymmetries increases public support. The treatment providing information about expected pollution reduction increases public support the most, especially for relatively ambitious designs.

A set of policy implications follow from our results. First, testing the waters to quantify the trade-offs between public support and efficiency for different designs is crucial, as it may reduce the risk that policymakers would make the wrong bet, and hence face a political failure that could prevent the implementation of a congestion charge for a long time. Second, providing information to voters, for instance drawing on the successful experience of other schemes, may contribute to close the gap between efficiency and public support. This is especially true for benefits that may not be immediately perceived by voters, such as improved air quality. The COVID-19 pandemic has, for instance, provided an excellent opportunity to policymakers around the world to convey to voters information about how their city and the sky above it would look like with fewer cars around. Third, identifying designs that can gather support among the local residents as well as commuters from the surrounding areas, and at the same time have a bite, may be especially hard. In line with standard public choice tenets, voters’ preferences tend to be very much driven by their own costs and benefits. Hence, depending on who has the right to say over the implementation of a congestion charge, public support can vary considerably. However, we identify several designs that reach and exceed majority support. In particular, public support is the highest among the residents of Geneva, who will be ultimately tasked with taking a decision. While this bodes well for the prospects of a congestion charge in Geneva, the variation in public support across designs should remind policymakers of the importance of carefully crafting legislation on potentially unpopular matters.

## Questionnaire

**General information**

Mobility demand in Geneva has been sharply increasing for decades. In the center of the agglomeration, around 1.5 millions trips per day are undertaken by around 520,000 vehicles.

A congestion charge aims at reducing traffic jams and traffic-related pollution by charging motorized vehicles circulating in a defined perimeter. The introduction of a congestion charge would encourage more efficient modes of transportation, reduce commuting times and air pollution, leading to a better use of the infrastructure. Coupled with other traffic management measures, the charge would allow to meet the goals of the law aiming for a coherent and balanced mobility, accepted by a large majority of Geneva’s voters in June 2016.

A congestion charge can be designed in different ways (perimeter, charge rate, use of revenues, exemptions etc.). The impacts on traffic, the environment, people’s purchasing power, and the revenues generated will depend on the specific design of the implemented congestion charge.

Several scenarios are currently under consideration. You have here the chance to express your preferences. Your responses will contribute to determine the interest in the introduction of a congestion charge in Geneva and under which conditions. In your answers, we will ask you to take into account all impacts of a possible congestion charge, which could be environmental, economic or social.

To facilitate your understanding, you will find in the next page a description of the possible characteristics of a congestion charge.

**Main characteristics of a congestion charge**

- **Perimeter**: As shown by the following map, the charging area could correspond either to the red perimeter (center) or the blue one (until the highway non included). Traffic on the federal highways is not affected by the congestion charge.
- **Charge level**: It would lie between a minimum of CHF 0.2 per passage through the cordon defining the perimeter and a maximum of CHF 5.-. The price would be charged both when entering and exiting the zone according to the modulation and exemptions.
- **Modulation**: The charge would be effective from Monday to Friday (6am-7pm), except on bank holidays. The price could vary in presence of pollution peaks (CHF 1.- top-up), according to the time of the day (CHF 1.50 top-up at peak-hours, 6:30am-9am and 4pm-7pm) or depending on the kilometers driven inside the perimeter (CHF 0.20 per kilometer driven). Alternatively, the charge could be effective only during peak hours (6:30am-9am and 4pm-7pm).
- **Use of revenues**: According to the first estimations, the congestion charge could generate revenues reaching CHF 50-100 million per year depending on the congestion charge characteristics. These revenues could be used in different ways: to finance public transportation in the Canton of Geneva (more frequency, enhanced quality, line extensions, lower off-peak fares), to finance a bridge or a tunnel to cross the Lake of Geneva, to adjust the vehicle registration fee in the Canton of Geneva, and to finance measures to reduce air and noise pollution.
- **Exemptions**: Emergency vehicles and those driven by disabled would not be subject to the congestion charge. Different exemption levels could be given to residents of the perimeter, scooters/motorbikes, electric vehicles, business deliveries, and frequent commuters (in the latter case, buying 200 passages would give a rebate on the following 200 passages).

**Instructions**

**Congestion treatment**

We would like to remind you that the goal of the congestion charge is to reduce congestion. In London and Milan, congestion decreased by 30% and 25%, respectively, following the implementation of a congestion charge. In Stockholm, traffic was reduced by more than 20%. We expect similar effects in Geneva.

**Pollution treatment**

We would like to remind you that the goal of the congestion charge is to reduce pollution and noise due to traffic. In London and Stockholm, small particles decreased by 10 to 15% and carbon dioxide by 13 to 16% following the implementation of a congestion charge. The decline in pollution has had a positive impact on public health. In addition to improvements in air quality, the level of noise declined as well. We expect similar effects in Geneva.

**Common information**

In what follows, you will vote 10 times on a congestion charge design. In each ballot, you have to choose among three alternatives: two scenarios with different congestion charges and the current situation without a congestion charge. During the vote, you will have access to further information in tooltips.

Please evaluate all proposition as if they would have been proposed by the local government and vote for your preferred option. There is no good or bad response.

Before the ballots, you will face an introductory question.

**Introductory question**

Which mode of transportation do you use most frequently (at least 4 times a week)?

- $$\square $$ Car
- $$\square $$ Bus,tramway
- $$\square $$ Train
- $$\square $$ Motorbike
- $$\square $$ Bicycle
- $$\square $$ Walking

**Vote**

If you would have to vote between these alternatives, which one would you prefer?

*Click on the underlined elements to get more information.*

**Table:**

Attributes						
	Perimeter	Charge rate	Modulation	Exemption	Beneficiaries	Revenues
Levels	Center	0.2	Constant	0%	Residents	Public transportation
	Ring	1	Peak hours only	25%	Motorbikes	Transport infrastructure
		2	Peak hours top-up	50%	Business deliveries	Pollution reduction
		3	Pollution top-up	75%	Electric vehicles	Tunnel or bridge
		4	Distance top-up	100%	Frequent commuters	Vehicle registration fee
		5				

**Tooltips content**

- **Perimeter**: The map in Fig. 1 appears.
- **Charge rate**: Drivers are charged both when entering and exiting the perimeter.
- **Peak hours only**: Drivers are charged only during peak hours (6.30 am to 9 am and 4 pm to 7 pm).
- **Peak hours top-up**: Drivers are charged from 6 am to 7 pm and there is a surcharge during peak hours (from 6.30 am to 9 am and from 4 pm to 7 pm).
- **Distance top-up**: Drivers are charged from 6 am to 7 pm and there is a surcharge of CHF 0.20 per kilometer driven within the perimeter.
- **Pollution top-up**: Drivers are charged from 6 am to 7 pm and there is a surcharge of CHF 1 when pollution is high.
- **Constant**: Drivers are charged from 6 am to 7 pm at a constant rate.
- **Frequent commuters**: The prepayment of 200 passages across the perimeter provides a discount on the following 200 passages.
- **Business deliveries**: Businesses with an economic activity within the perimeter can benefit from an exemption.
- **No exemption**: No exemption is granted, except for emergency vehicles and disabled individuals.
- **Public transportation**: Revenues earmarked for public transportation in the Canton of Geneva (*Transports Publics Genevois*) with the objective to improve quality, frequency, and coverage, as well as to lead to lower fares during off-peak times.
- **Tunnel or bridge**: Revenues earmarked for a tunnel or bridge crossing the Lake Geneva (*Grande Traversée du Lac*) as well as for accompanying measures to manage road traffic in the center of the agglomeration.
- **Transport infrastructure**: Revenues earmarked for improvements in transport supply such as the road network and the cycling lanes.
- **Pollution**: Revenues earmarked for measures abating air and noise pollution such as sound-absorbing coating.
- **Vehicle registration fee**: Revenues redistributed to the population of the Canton of Geneva via a reduction in the annual vehicle registration fee.

**Mobility equipment**

Here, we ask you some questions about your equipment in transportation passes and in cars.

1. Do you have a driving license allowing you to drive a car?
  - $$\square $$ Yes
  - $$\square $$ No
2. How many cars do you have in your household? Take also into account company cars that are always at your disposal.
  - _ Car(s)
3. Do you have the possibility to borrow a car from a relative or your family?
  - $$\square $$ Yes
  - $$\square $$ No
4. Do you have a monthly or annual pass for the Swiss public transportation system (except the Half Fare Travelcard) or the local mass transit system (TPG, Unireso, etc)?
  - $$\square $$ Yes
  - $$\square $$ No

**Your trips to Geneva**

To better know your mobility habits we ask you some questions about your trips to and from the center of Geneva.

1. What is your municipality of residence?
2. What is your current activity?
  - $$\square $$ Student
  - $$\square $$ At home
  - $\square$ Full-time or part-time worker
  - $$\square $$ Job seeking
  - $$\square $$ Retiree
3. In which municipality do you work/study?
4. Which mode of transportation do you mainly use to commute to your working or studying place? Only one response possible. If you use several transportation modes for this journey, indicate the one that you use on the last part of the trip.
  - $$\square $$ Car
  - $\square$ Mass transit (bus/tramway)
  - $$\square $$ Train
  - $$\square $$ Motorbike
  - $$\square $$ Bicycle
  - $$\square $$ Walking
5. What is the average duration of your trip from home to your working place or from home to your studying place (one-way) in minutes?
  - _ minutes
6. At what time do you leave your residency in general for this trip?
7. When commuting to your working or studying place, do you cross the municipalities of Geneva, Lancy or Carouge ?
  - $$\square $$ Yes
  - $$\square $$ No
8. Are you sometimes commuting to your working place with another mode of transportation?
  - $$\square $$ Yes
  - $$\square $$ No
9. If yes, which one? Only one response possible. If you use several transportation modes for this journey, indicate the one that you use on the last part of the trip.
  - $$\square $$ Car
  - $$\square $$ Mass transit
  - $$\square $$ Train
  - $$\square $$ Motorbikes
  - $$\square $$ Bicycle
  - $$\square $$ Walking
10. What is the average length of this trip? (in minutes)
11. At what time do you leave in general your residency for this trip?
12. Is it possible for you to commute by mass transit to your working place?
  - $$\square $$ Yes
    $$\square $$ No
13. What is the average duration of this trip by mass transit? (in minutes)
  - _ minutes
14. Do you sometimes, during the week (Monday to Friday), cross the municipalities of Geneva, Carouge or Lancy by car for shopping or leisure activities (visiting friends or family, restaurants, sport, etc.)?
  - $$\square $$ Yes
    $$\square $$ No
15. Do you sometimes leave your municipality of residence by car for shopping or leisure activities (visiting friends or family, restaurants,sport, etc) during the week (Monday to Friday)?
  - $$\square $$ Yes
    $$\square $$ No
16. Could you indicate the destination of the most recent trip that you undertook by car for shopping or leisure activities?
17. How long was this trip? (in minutes)
  - _ minutes
18. At what time did you leave your residence for this trip?

**Respondent profile**

1. Are you?
  - $$\square $$ A female
    $$\square $$ A male
2. How old are you?
  - _ years old
3. How many people usually live in your household, included you? (Include your family, but also any person living at least 4 days a week in your household)
  - _ Adults _ Children (0-18 years)
4. What is the last diploma that you obtained?
  - $\square$ Compulsory school certificate, no diploma, primary school certificate
  - $$\square $$ Apprenticeship
  - $\square$ Post-compulsory school : secondary level, high school
  - $\square$ Diploma of higher education (DEUG, DUT, BTS)
  - $\square$ University degree (undergraduate, master, PhD) : university, institute of technology, and university of applied sciences.
5. What is the total monthly gross income (including benefits and other subsidies) of your household, taking into account the income of all the members of the household? (In Euros/CHF)
  - $$\square $$ Less than 900
  - $$\square $$ From 901 to 1 500
  - $$\square $$ From 1 501 to 2 000
  - $$\square $$ From 2 001 to 3 000
  - $$\square $$ From 3 001 to 4 000
  - $$\square $$ From 4 001 to 5 000
  - $$\square $$ From 5 001 to 6 000
  - $$\square $$ From 6 001 to 7 000
  - $$\square $$ From 7 001 to 8 000
  - $$\square $$ From 8 001 to 9 000
  - $$\square $$ From 9 001 to 10 000
  - $$\square $$ From 10 001 to 11 000
  - $$\square $$ From 11 001 to 12 000
  - $$\square $$ More than 12 000
  - $\square$ I do not want to answer.

## Tables

**Table 8:** Summary information available at any time to respondents

Variable	Description
Perimeter	The map in Fig. 1 appears.
Charge rate	Drivers are charged both when entering and exiting the perimeter.
Peak hours only	Drivers are charged only during peak hours (6.30 am to 9 am and 4 pm to 7 pm).
Peak hours top-up	Drivers are charged from 6 am to 7 pm and there is a surcharge during peak hours (from 6.30 am to 9 am and from 4 pm to 7 pm).
Distance top-up	Drivers are charged from 6 am to 7 pm and there is a surcharge of CHF 0.20 per kilometer driven within the perimeter.
Pollution top-up	Drivers are charged from 6 am to 7 pm and there is a surcharge of CHF 1 when pollution is high.
Constant	Drivers are charged from 6 am to 7 pm at a constant rate.
Frequent commuters	The prepayment of 200 passages across the perimeter provides a discount on the following 200 passages.
Business deliveries	Businesses with an economic activity within the perimeter can benefit from an exemption.
No exemption	No exemption is granted, except for emergency vehicles and disabled individuals.
Public transportation	Revenues earmarked for public transportation in the Canton of Geneva (*Transports Publics Genevois*) with the objective of improving quality, frequency, and coverage, as well as to lead to lower fares during off-peak times.
Tunnel or bridge	Revenues earmarked for a tunnel or bridge crossing the Lake Geneva (*Grande Traversée du Lac*) as well as for accompanying measures to manage road traffic in the center of the agglomeration.
Transport infrastructure	Revenues earmarked for improvements in transport infrastructure such as the road network and cycling lanes.
Pollution	Revenues earmarked for measures abating air and noise pollution such as sound-absorbing coating.
Vehicle registration fee	Revenues redistributed to the population of the Canton of Geneva via a reduction in the annual vehicle registration fee.

### Sample Characteristics and Representativity

**Table 9:** Sample composition

Variable	Mean	Std. Dev	Min	Max	N
Gender (female)	0.512	0.500	0	1	1414
Age	41.789	13.738	18	77	1414
Household size	2.252	1.677	0	10	1414
Number of cars in household	1.405	0.752	0	5	1171
Public transportation pass holder	0.500	0.500	0	1	1222
*Household monthly income*					
< CHF 900	0.023	0.151	0	1	995
CHF 901 - CHF 1,500	0.021	0.144	0	1	995
CHF 1,501 - CHF 2,000	0.02	0.139	0	1	995
CHF 2,001 - CHF 3,000	0.037	0.188	0	1	995
CHF 3,001 - CHF 4,000	0.062	0.242	0	1	995
CHF 4,001 - CHF 5,000	0.070	0.255	0	1	995
CHF 5,001 - CHF 6,000	0.079	0.269	0	1	995
CHF 6,001 - CHF 7,000	0.062	0.240	0	1	995
CHF 7,001 - CHF 8,000	0.050	0.219	0	1	995
CHF 8,001 - CHF 9,000	0.057	0.231	0	1	995
CHF 9,001 - CHF 10,000	0.053	0.224	0	1	995
CHF 10,001 - CHF 11,000	0.039	0.193	0	1	995
CHF 11,001 - CHF 12,000	0.033	0.178	0	1	995
> CHF 12,000	0.099	0.299	0	1	995
*Education level*					
Compulsory schooling	0.065	0.247	0	1	1398
Apprenticeship	0.199	0.4	0	1	1398
Post-compulsory school	0.255	0.436	0	1	1398
Superior first cycle degree	0.107	0.309	0	1	1398
Superior second cycle degree	0.362	0.481	0	1	1398
*Residence area*					
Switzerland	0.714	0.452	0	1	1414
Canton of Geneva	0.553	0.497	0	1	1414
Canton of Vaud	0.161	0.367	0	1	1414
France	0.286	0.452	0	1	1414
Genevois	0.039	0.193	0	1	1414
Gex	0.127	0.333	0	1	1414
Haute-Savoie	0.031	0.174	0	1	1414
Annemasse agglomeration	0.089	0.285	0	1	1414
*Commuting*					
Car	0.466	0.499	0	1	999
Bus and tramway	0.246	0.431	0	1	999
Train	0.091	0.288	0	1	999
Motorcycle	0.079	0.270	0	1	999
Bicycle	0.050	0.218	0	1	999
Walking	0.067	0.250	0	1	999

**Table 10:** Balance of covariates

Variable	Congestion			Pollution			Control				
	Mean	Std. Dev	N	Mean	Std. Dev	N	Mean	Std. Dev	N	Min	Max
Gender (female)	0.52	0.50	454	0.54**	0.50	478	0.47	0.50	482	0	1
Age	41.13	13.67	454	41.86	14.30	478	42.34	13.19	482	18	77
Household size	2.29	1.62	454	2.28	1.70	478	2.19	1.70	482	0	10
Number of cars in household	1.39	0.69	374	1.38	0.75	392	1.44	0.80	405	0	5
Public transportation pass holder	0.48	0.50	389	0.49	0.50	407	0.53	0.50	426	0	1
*Household monthly income*											
< CHF 900	0.03	0.17	454	0.02	0.13	478	0.02	0.16	482	0	1
CHF 901 - CHF 1,500	0.02	0.15	454	0.02	0.15	478	0.02	0.14	482	0	1
CHF 1,501 - CHF 2,000	0.02	0.13	454	0.02	0.14	478	0.02	0.15	482	0	1
CHF 2,001 - CHF 3,000	0.02*	0.15	454	0.05	0.22	478	0.04	0.19	482	0	1
CHF 3,001 - CHF 4,000	0.07	0.25	454	0.05	0.21	478	0.07	0.26	482	0	1
CHF 4,001 - CHF 5,000	0.07	0.25	454	0.08	0.27	478	0.06	0.24	482	0	1
CHF 5,001 - CHF 6,000	0.09	0.29	454	0.06	0.24	478	0.08	0.27	482	0	1
CHF 6,001 - CHF 7,000	0.05	0.22	454	0.06	0.24	478	0.07	0.25	482	0	1
CHF 7,001 - CHF 8,000	0.05	0.22	454	0.05	0.22	478	0.05	0.22	482	0	1
CHF 8,001 - CHF 9,000	0.05	0.21	454	0.06	0.25	478	0.06	0.23	482	0	1
CHF 9,001 - CHF 10,000	0.05	0.22	454	0.04**	0.20	478	0.07	0.25	482	0	1
CHF 10,001 - CHF 11,000	0.05	0.21	454	0.03	0.16	478	0.04	0.20	482	0	1
CHF 11,001 - CHF 12,000	0.03	0.17	454	0.03	0.17	478	0.04	0.18	482	0	1
> CHF 12,000	0.12	0.32	454	0.09	0.29	478	0.09	0.28	482	0	1
*Education level*											
Compulsory schooling	0.05*	0.21	454	0.06	0.24	478	0.08	0.28	482	0	1
Apprenticeship	0.19	0.39	454	0.20	0.40	478	0.21	0.40	482	0	1
Post-compulsory school	0.26	0.44	454	0.28*	0.45	478	0.23	0.42	482	0	1
Superior first cycle degree	0.10	0.30	454	0.09	0.29	478	0.12	0.33	482	0	1
Superior second cycle degree	0.39	0.49	454	0.34	0.47	478	0.35	0.48	482	0	1
*Residence area*											
Switzerland	0.71	0.45	454	0.72	0.45	478	0.71	0.45	482	0	1
Canton of Geneva	0.55	0.50	454	0.55	0.50	478	0.56	0.50	482	0	1
Canton of Vaud	0.17	0.37	454	0.17	0.37	478	0.15	0.36	482	0	1
France	0.29	0.45	454	0.28	0.45	478	0.29	0.45	482	0	1
Genevois	0.03	0.17	454	0.04	0.20	478	0.05	0.21	482	0	1
Gex	0.13	0.34	454	0.13	0.34	478	0.12	0.33	482	0	1
Haute-Savoie	0.04	0.20	454	0.02	0.14	478	0.03	0.18	482	0	1
Annemasse agglomeration	0.09	0.28	454	0.09	0.29	478	0.09	0.29	482	0	1
*Commuting*											
Car	0.51	0.50	319	0.43	0.50	337	0.46	0.50	343	0	1
Bus and tramway	0.22	0.41	319	0.28	0.45	337	0.24	0.43	343	0	1
Train	0.09	0.28	319	0.10	0.30	337	0.09	0.28	343	0	1
Motorcycle	0.08	0.27	319	0.06	0.24	337	0.09	0.29	343	0	1
Bicycle	0.04	0.20	319	0.05	0.22	337	0.06	0.23	343	0	1
Walking	0.06	0.24	319	0.08	0.27	337	0.06	0.24	343	0	1

*$$p<0.1$$, **$$p<0.05$$, ***$$p<0.01$$

**Table 11:** Socioeconomic characteristics of the underlying population: Geneva

Variable	Geneva	Sample
	Mean	Mean
Gender (female)	0.515	0.567
Number of cars per inhabitant	0.448	0.635
*Education level*		
Compulsory schooling	0.286	0.31
Secondary education	0.326	0.353
Tertiary education	0.388	0.326
*Commuting*		
Car and motorcycle	0.45	0.434

*Source*: All variables come from the Cantonal Office of Statistics

**Table 12:** Socioeconomic characteristics of the underlying population - entire metropolitan area (“Grand-Genève”)

Variable	Grand-Genève	Sample
	Mean	Mean
Gender (female)	0.513	0.512
Number of cars per inhabitant	0.533	0.687
*Education level*		
Compulsory schooling	0.205	0.265
Secondary education	0.349	0.362
Tertiary education	0.439	0.362
*Residence area*		
Switzerland	0.65	0.714
France	0.35	0.286
*Commuting*		
Car and motorcycle	0.57	0.545

*Source*: All variables come from the Cross-Border Observatory for Statistics and the Swiss Federal Office of Statistics

### Additional empirical results

**Table 13:** Estimates from conditional logit: residents of the perimeter, living in the Canton of Geneva but outside the perimeters, not living in the Canton of Geneva

	(1)		(2)		(3)	
	Residents		Living in the Canton of Geneva but outside the perimeters		Not living in the Canton of Geneva	
*Charge rate*						
CHF 0 (reference)						
CHF 0.2	$-$0.053*	(0.028)	$-$0.088**	(0.042)	$-$0.034	(0.025)
CHF 1	$-$0.072**	(0.029)	$-$0.150***	(0.039)	$-$0.119***	(0.025)
CHF 2	$-$0.144***	(0.029)	$-$0.231***	(0.039)	$-$0.220***	(0.024)
CHF 3	$-$0.215***	(0.028)	$-$0.264***	(0.038)	$-$0.296***	(0.025)
CHF 4	$-$0.210***	(0.029)	$-$0.302***	(0.038)	$-$0.340***	(0.024)
CHF 5	$-$0.268***	(0.029)	$-$0.336***	(0.038)	$-$0.375***	(0.025)
*Perimeter*						
Center (reference)						
Ring	$-$0.005	(0.012)	$-$0.008	(0.021)	$-$0.02*	(0.011)
*Modulation*						
Constant (reference)						
Peak hours only	$-$0.002	(0.014)	0.034	(0.024)	0.029**	(0.014)
Peak hours top-up	$-$0.018	(0.014)	0.041*	(0.022)	0.017	(0.014)
Distance top-up	$-$0.081***	(0.016)	$-$0.056**	(0.024)	$-$0.009	(0.015)
Pollution top-up	$-$0.036**	(0.014)	0.032	(0.022)	0.001	(0.014)
*Beneficiaries*						
Business deliveries (reference)						
Residents	0.068***	(0.016)	0.045*	(0.024)	$-$0.014	(0.014)
Motorbikes	$-$0.006	(0.016)	0.010	(0.024)	$-$0.035**	(0.015)
Frequent commuters	0.021	(0.016)	0.027	(0.024)	0.006	(0.014)
Electric vehicles	0.012	(0.015)	$-$0.037	(0.026)	$-$0.05***	(0.016)
*Exemption level*						
0% (reference)						
25%	0.01	(0.016)	$-$0.020	(0.021)	0.004	(0.014)
50%	0.039***	(0.015)	0.029	(0.022)	0.036**	(0.014)
75%	0.039**	(0.016)	0.048**	(0.021)	0.049***	(0.014)
100%	0.054***	(0.016)	0.026	(0.023)	0.098***	(0.015)
*Use of revenue*						
Vehicle registration fee (reference)						
Public transportation	0.087***	(0.016)	0.079***	(0.025)	0.080***	(0.015)
Transport infrastructure	0.058***	(0.015)	0.052**	(0.023)	0.044***	(0.015)
Pollution reduction	0.066***	(0.016)	0.053**	(0.024)	0.039**	(0.016)
Tunnel or bridge	0.020	(0.016)	0.038	(0.027)	0.050***	(0.016)
Number of respondents	547		235		632	
Number of observations	16,407		7,047		18,954	
Pseudo $R^{2}$	0.0456		0.0775		0.1148	

Estimates report average marginal effects from conditional logit

Heteroskedasticity-robust standard errors in parentheses

Continuous *p*-values are provided in Table 24

*$$p<0.1$$, **$$p<0.05$$, ***$$p<0.01$$

**Table 14:** Estimates from conditional logit by commuting mode

	(1)		(2)	
	Car and motorbike		Public transportation, cycling and walking	
*Charge rate*				
CHF 0 (reference)				
CHF 0.2	$-$0.043	(0.027)	$-$0.056**	(0.022)
CHF 1	$-$0.140***	(0.027)	$-$0.086***	(0.022)
CHF 2	$-$0.248***	(0.026)	$-$0.159***	(0.022)
CHF 3	$-$0.321***	(0.026)	$-$0.224***	(0.022)
CHF 4	$-$0.328***	(0.026)	$-$0.252***	(0.022)
CHF 5	$-$0.388***	(0.027)	$-$0.292***	(0.022)
*Perimeter*				
Center (reference)				
Ring	$-$0.018	(0.012)	$-$0.004	(0.009)
*Modulation*				
Constant (reference)				
Peak hours only	0.031**	(0.015)	0.010	(0.011)
Peak hours top-up	0.014	(0.015)	0.004	(0.012)
Distance top-up	$-$0.012	(0.016)	$-$0.063***	(0.012)
Pollution top-up	0.004	(0.015)	$-$0.017	(0.011)
*Beneficiaries*				
Business deliveries (reference)				
Residents	$-$0.006	(0.016)	0.047***	(0.012)
Motorbikes	$-$0.016	(0.016)	$-$0.017	(0.013)
Frequent commuters	0.009	(0.015)	0.018	(0.012)
Electric vehicles	$-$0.067***	(0.018)	0.002	(0.012)
*Exemption level*				
0% (reference)				
25%	0.020	(0.015)	$-$0.007	(0.012)
50%	0.045***	(0.015)	0.031***	(0.012)
75%	0.061***	(0.015)	0.036***	(0.012)
100%	0.096***	(0.016)	0.054***	(0.013)
*Use of revenue*				
Vehicle registration fee (reference)				
Public transportation	0.061***	(0.016)	0.093***	(0.013)
Transport infrastructure	0.033**	(0.015)	0.062***	(0.012)
Pollution reduction	0.051***	(0.015)	0.052***	(0.013)
Tunnel or bridge	0.05***	(0.016)	0.027**	(0.013)
Number of respondents	544		870	
Number of observations	16,314		26,094	
Pseudo $R^{2}$	0.1206		0.0552	

Estimates report average marginal effects from conditional logit

Heteroscedasticity-robust standard errors in parentheses

Continuous *p*-values are provided in Table 25

*$$p<0.1$$, **$$p<0.05$$, ***$$p<0.01$$

**Table 15:** Estimates from conditional logit by commuting frequency

	(1)		(2)		(3)	
	Inhabitants of the Canton of Geneva		6-7 trips/week to Geneva		1-5 trips/week to Geneva	
*Charge rate*						
CHF 0 (reference)						
CHF 0.2	$-$0.046	(0.029)	$-$0.103***	(0.036)	$-$0.03	(0.026)
CHF 1	$-$0.0742***	(0.029)	$-$0.151***	(0.037)	$-$0.119***	(0.026)
CHF 2	$-$0.151***	(0.029)	$-$0.255***	(0.036)	$-$0.201***	(0.026)
CHF 3	$-$0.214***	(0.029)	$-$0.288***	(0.034)	$-$0.290***	(0.026)
CHF 4	$-$0.205***	(0.029)	$-$0.322***	(0.034)	$-$0.335***	(0.026)
CHF 5	$-$0.266***	(0.028)	$-$0.340***	(0.036)	$-$0.383***	(0.026)
*Perimeter*						
Center (reference)						
Ring	0.002	(0.012)	0.006	(0.015)	$-$0.026**	(0.011)
*Modulation*						
Constant (reference)						
Peak hours only	$-$0.008	(0.014)	0.038**	(0.019)	0.031**	(0.014)
Peak hours top-up	$-$0.01	(0.015)	0.017	(0.019)	0.02	(0.014)
Distance top-up	$-$0.084***	(0.016)	$-$0.055***	(0.020)	$-$0.004	(0.016)
Pollution top-up	$-$0.024*	(0.015)	$-$0.019	(0.021)	0.013	(0.014)
*Beneficiaries*						
Business deliveries (reference)						
Residents	0.053***	(0.016)	0.031	(0.02)	0.003	(0.015)
Motorbikes	$-$0.009	(0.017)	0.013	(0.021)	$-$0.04**	(0.016)
Frequent commuters	0.021	(0.016)	0.041**	(0.021)	$-$0.003	(0.015)
Electric vehicles	$-$0.011	(0.016)	$-$0.007	(0.020)	$-$0.043**	(0.017)
*Exemption level*						
0% (reference)						
25%	$-$0.001	(0.015)	$-$0.0002	(0.020)	0.007	(0.015)
50%	0.025	(0.015)	0.042**	(0.018)	0.043***	(0.016)
75%	0.026	(0.016)	0.065***	(0.021)	0.050***	(0.014)
100%	0.041**	(0.016)	0.045**	(0.021)	0.107***	(0.015)
*Use of revenue*						
Vehicle registration fee (reference)						
Public transportation	0.076***	(0.016)	0.106***	(0.022)	0.075***	(0.016)
Transport infrastructure	0.045***	(0.015)	0.060***	(0.022)	0.051***	(0.015)
Pollution reduction	0.054***	(0.016)	0.062***	(0.022)	0.044***	(0.016)
Tunnel or bridge	0.007	(0.016)	0.054**	(0.023)	0.052***	(0.017)
Number of respondents	515		314		585	
Number of observations	15,444		9,414		17,550	
Pseudo $R^{2}$	0.0531		0.0735		0.1057	

Estimates report average marginal effects from conditional logit

Heteroscedasticity-robust standard errors in parentheses

Continuous *p*-values are provided in Table 26

*$$p<0.1$$, **$$p<0.05$$, ***$$p<0.01$$

**Table 16:** Estimates from conditional logit: cars vs. motorbikes

	(1)		(2)	
	Car		Motorbike	
*Charge rate*				
CHF 0 (reference)				
CHF 0.2	− 0.022	(0.029)	− 0.159**	(0.069)
CHF 1	− 0.124***	(0.028)	− 0.234***	(0.072)
CHF 2	− 0.233***	(0.028)	− 0.334***	(0.065)
CHF 3	− 0.319***	(0.028)	− 0.343***	(0.069)
CHF 4	− 0.321***	(0.029)	− 0.376***	(0.064)
CHF 5	− 0.375***	(0.029)	− 0.454***	(0.068)
*Perimeter*				
Center (reference)				
Ring	− 0.02	(0.013)	− 0.008	(0.032)
*Modulation*				
Constant (reference)				
Peak hours only	0.029*	(0.016)	0.05	(0.037)
Peak hours top-up	0.013	(0.016)	0.028	(0.039)
Distance top-up	− 0.013	(0.018)	0.008	(0.039)
Pollution top-up	0.004	(0.016)	0.013	(0.039)
*Beneficiaries*				
Business deliveries (reference)				
Resident	− 0.004	(0.017)	− 0.008	(0.035)
Motorbikes	− 0.048***	(0.017)	0.147***	(0.037)
Frequent commuters	0.013	(0.017)	− 0.019	(0.038)
Electric vehicles	− 0.073***	(0.02)	− 0.026	(0.04)
*Exemption level*				
0% (reference)				
25%	0.011	(0.017)	0.071*	(0.04)
50%	0.037**	(0.017)	0.081**	(0.04)
75%	0.052***	(0.017)	0.098***	(0.034)
100%	0.092***	(0.017)	0.117***	(0.038)
*Use of revenue*				
Vehicle registration fee (reference)				
Public transportation	0.053***	(0.017)	0.101***	(0.038)
Transport infrastructure	0.036**	(0.017)	0.009	(0.042)
Pollution reduction	0.054***	(0.016)	0.029	(0.041)
Tunnel or bridge	0.045**	(0.018)	0.085**	(0.043)
Number of respondents	465		79	
Number of observations	13,944		2,370	
Pseudo $R^{2}$	0.1285		0.1066	

Estimates report average marginal effects from conditional logit

Heteroscedasticity-robust standard errors in parentheses

Continuous *p*-values are provided in Table 27

*$$p<0.1$$, **$$p<0.05$$, ***$$p<0.01$$

### Tables displaying coefficients for control variables

**Table 17:** The impact of informational treatments on public support, for each charge rate

	(1)	(2)	(3)	(4)	(5)	(6)
	CHF 0.2	CHF 1	CHF 2	CHF 3	CHF 4	CHF 5
*Treatments*						
Control (reference)						
Congestion	0.015	0.022	$-$0.001	0.006	0.003	0.016
	(0.017)	(0.017)	(0.016)	(0.015)	(0.015)	(0.014)
Pollution	0.006	0.016	0.039**	0.036**	0.043***	0.037***
	(0.017)	(0.017)	(0.016)	(0.014)	(0.014)	(0.013)
*Control variables*						
Gender (female)	$-$0.004	0.011	$-$0.004	0.014	$-$0.006	$-$0.004
	(0.015)	(0.015)	(0.014)	(0.013)	(0.012)	(0.011)
Age	$-$0.003***	$-$0.002***	$-$0.002***	$-$0.001	$-$0.002***	$-$0.001*
	(0.001)	(0.001)	(0.001)	(0.001)	(0.0005)	(0.000)
Household size	0.011**	0.016***	0.012***	0.010***	0.008**	0.012***
	(0.005)	(0.005)	(0.004)	(0.004)	(0.004)	(0.003)
*Number of cars in household*						
No car (reference)						
1 car	$-$0.023	0.008	$-$0.023	0.035	0.009	0.007
	(0.031)	(0.031)	(0.027)	(0.026)	(0.024)	(0.023)
2 cars	$-$0.026	0.018	$-$0.030	0.021	$-$0.005	$-$0.009
	(0.033)	(0.033)	(0.030)	(0.028)	(0.027)	(0.025)
3 cars	0.122**	0.015	$-$0.029	0.025	$-$0.041	$-$0.099**
	(0.047)	(0.046)	(0.043)	(0.041)	(0.041)	(0.042)
4 cars	$-$0.154	0.034	$-$0.070	$-$0.005	$-$0.049	0.102*
	(0.095)	(0.087)	(0.084)	(0.077)	(0.079)	(0.054)
5 cars	$-$0.174	$-$0.090	$-$0.210	$-$0.064	$-$0.054	0.000
	(0.187)	(0.184)	(0.205)	(0.171)	(0.163)	(.)
No answer	$-$0.079*	$-$0.064	$-$0.017	0.038	$-$0.009	$-$0.003
	(0.047)	(0.047)	(0.042)	(0.038)	(0.038)	(0.036)
*Public transportation pass holder*						
No (reference)						
Yes	0.090***	0.068***	0.046***	0.045***	0.076***	0.034**
	(0.018)	(0.018)	(0.016)	(0.015)	(0.015)	(0.014)
No answer	0.098**	0.092**	0.016	0.022	0.070*	0.049
	(0.046)	(0.046)	(0.041)	(0.037)	(0.038)	(0.036)
*Household monthly income:*						
< CHF 900 (reference)						
CHF 901 - CHF 1,500	0.058	$-$0.102	$-$0.026	0.046	$-$0.023	$-$0.169***
	(0.067)	(0.069)	(0.063)	(0.054)	(0.057)	(0.056)
CHF 1,501 - CHF 2,000	0.128*	0.047	0.043	0.083	0.053	$-$0.019
	(0.067)	(0.065)	(0.061)	(0.053)	(0.053)	(0.042)
CHF 2,001 - CHF 3,000	0.078	0.119**	0.109**	0.047	0.064	$-$0.054
	(0.060)	(0.057)	(0.052)	(0.046)	(0.046)	(0.039)
CHF 3,001 - CHF 4,000	0.102*	0.062	$-$0.008	0.003	$-$0.013	$-$0.064*
	(0.056)	(0.053)	(0.050)	(0.044)	(0.044)	(0.036)
CHF 4,001 - CHF 5,000	0.108*	0.048	$-$0.004	$-$0.019	$-$0.029	$-$0.112***
	(0.055)	(0.053)	(0.049)	(0.043)	(0.043)	(0.036)
CHF 5,001 - CHF 6,000	0.124**	0.025	0.012	$-$0.051	$-$0.040	$-$0.187***
	(0.054)	(0.052)	(0.048)	(0.043)	(0.042)	(0.037)
CHF 6,001 - CHF 7,000	0.087	$-$0.002	0.016	$-$0.022	$-$0.011	$-$0.088**
	(0.056)	(0.054)	(0.050)	(0.044)	(0.043)	(0.036)
CHF 7,001 - CHF 8,000	0.119**	0.030	0.039	0.002	0.004	$-$0.099***
	(0.057)	(0.055)	(0.051)	(0.045)	(0.044)	(0.037)
CHF 8,001 - CHF 9,000	0.051	$-$0.060	$-$0.032	$-$0.012	$-$0.009	$-$0.101***
	(0.058)	(0.056)	(0.051)	(0.044)	(0.044)	(0.037)
CHF 9,001 - CHF 10,000	0.096*	0.053	0.036	$-$0.004	0.025	$-$0.137***
	(0.058)	(0.055)	(0.051)	(0.044)	(0.044)	(0.038)
CHF 10,001 - CHF 11,000	0.056	$-$0.013	0.012	$-$0.036	0.001	$-$0.120***
	(0.061)	(0.059)	(0.054)	(0.048)	(0.047)	(0.041)
CHF 11,001 - CHF 12,000	0.073	$-$0.076	$-$0.034	$-$0.045	$-$0.042	$-$0.143***
	(0.063)	(0.063)	(0.058)	(0.051)	(0.050)	(0.042)
> CHF 12,000	0.154***	0.018	$-$0.009	$-$0.034	$-$0.016	$-$0.133***
	(0.054)	(0.052)	(0.049)	(0.043)	(0.042)	(0.035)
No answer	0.110**	0.010	$-$0.005	$-$0.075*	$-$0.028	$-$0.134***
	(0.050)	(0.047)	(0.044)	(0.039)	(0.038)	(0.031)
*Education leve*l						
Compulsory schooling (reference)						
Apprenticeship	0.005	0.013	$-$0.023	$-$0.014	$-$0.060**	$-$0.018
	(0.032)	(0.032)	(0.029)	(0.028)	(0.026)	(0.024)
Post-compulsory school	0.054*	0.014	0.000	$-$0.009	$-$0.059**	$-$0.006
	(0.030)	(0.030)	(0.028)	(0.027)	(0.024)	(0.023)
Superior first cycle degree	0.031	0.003	0.008	$-$0.012	$-$0.062**	0.001
	(0.035)	(0.035)	(0.032)	(0.031)	(0.029)	(0.027)
Superior second cycle degree	0.002	$-$0.007	$-$0.014	0.012	$-$0.040*	0.015
	(0.030)	(0.030)	(0.028)	(0.026)	(0.024)	(0.023)
No answer	$-$0.406***	$-$0.255***	$-$0.090	0.057	0.057	0.084*
	(0.114)	(0.095)	(0.072)	(0.055)	(0.050)	(0.050)
*Residence area*						
Canton of Geneva (reference)						
Canton of Vaud	$-$0.104***	$-$0.068***	$-$0.009	0.015	$-$0.018	0.012
	(0.023)	(0.023)	(0.020)	(0.019)	(0.018)	(0.016)
Genevois	0.137***	0.062*	$-$0.030	$-$0.041	$-$0.102***	$-$0.104***
	(0.036)	(0.037)	(0.036)	(0.034)	(0.038)	(0.036)
Gex	0.069***	0.033	$-$0.034	$-$0.061***	$-$0.065***	$-$0.061***
	(0.023)	(0.023)	(0.022)	(0.022)	(0.021)	(0.020)
Haute-Savoie	0.263***	0.162***	0.009	$-$0.073*	$-$0.209***	$-$0.144***
	(0.041)	(0.039)	(0.040)	(0.042)	(0.057)	(0.048)
Annemasse agglomeration	0.137***	0.062**	0.009	$-$0.024	$-$0.041*	$-$0.036*
	(0.026)	(0.026)	(0.024)	(0.023)	(0.022)	(0.020)
*Commuting*						
Car (reference)						
Bus and tramway	$-$0.065***	0.027	0.044**	0.060***	$-$0.007	0.033*
	(0.024)	(0.024)	(0.021)	(0.020)	(0.019)	(0.018)
Train	0.012	0.046	0.022	0.013	$-$0.023	$-$0.007
	(0.034)	(0.034)	(0.030)	(0.028)	(0.028)	(0.025)
Motorcycle	$-$0.027	0.001	$-$0.010	0.069**	0.020	0.011
	(0.034)	(0.033)	(0.031)	(0.027)	(0.026)	(0.026)
Bicycle	$-$0.017	$-$0.048	0.065*	0.053	0.048	0.090***
	(0.038)	(0.042)	(0.037)	(0.033)	(0.031)	(0.027)
Walking	$-$0.072*	0.031	0.070**	0.061**	0.015	0.009
	(0.037)	(0.036)	(0.033)	(0.029)	(0.028)	(0.027)
No answer	$-$0.022	0.034	0.020	0.029	$-$0.014	$-$0.007
	(0.024)	(0.023)	(0.022)	(0.020)	(0.020)	(0.019)
Number of respondents	1414	1414	1414	1414	1414	1414
Number of observations	4,736	4,711	4,702	4,702	4,711	4,702
$R^{2}$	0.0432	0.0265	0.0202	0.0290	0.0412	0.0462

Estimates report average marginal effects from logit with control variables

Heteroskedasticity-robust standard errors in parentheses

*$$p<0.1$$, **$$p<0.05$$, ***$$p<0.01$$

**Table 18:** The impact of informational treatments on public support, for each modulation

	(1)	(2)	(3)	(4)	(5)
	Constant	Peak hours only	Peak hours top-up	Distance top-up	Pollution top-up
*Treatments*					
Control (reference)					
Congestion	0.020	$-$0.005	0.005	0.026*	0.006
	(0.015)	(0.015)	(0.015)	(0.014)	(0.015)
Pollution	0.018	0.021	0.047***	0.021	0.045***
	(0.015)	(0.015)	(0.014)	(0.014)	(0.014)
*Control variables*					
Gender (female)	$-$0.012	0.014	$-$0.015	0.011	0.010
	(0.012)	(0.013)	(0.013)	(0.012)	(0.012)
Age	$-$0.001***	$-$0.002***	$-$0.001***	$-$0.001**	$-$0.002***
	(0.001)	(0.001)	(0.001)	(0.000)	(0.001)
Household size	0.013***	0.009**	0.014***	0.011***	0.010***
	(0.004)	(0.004)	(0.004)	(0.004)	(0.004)
*Number of cars in household*					
No car (reference)					
1 car	0.007	0.024	$-$0.031	$-$0.005	0.013
	(0.026)	(0.027)	(0.026)	(0.025)	(0.026)
2 cars	$-$0.038	0.037	$-$0.030	$-$0.010	0.015
	(0.028)	(0.029)	(0.028)	(0.027)	(0.028)
3 cars	$-$0.014	$-$0.012	0.004	$-$0.004	0.038
	(0.040)	(0.041)	(0.039)	(0.038)	(0.039)
4 cars	0.022	$-$0.121	$-$0.021	0.041	$-$0.052
	(0.069)	(0.090)	(0.072)	(0.072)	(0.077)
5 cars	$-$0.006	0.107	0.000	0.000	$-$0.211
	(0.130)	(0.144)	(.)	(.)	(0.202)
No answer	$-$0.055	$-$0.030	$-$0.003	$-$0.029	0.001
	(0.040)	(0.041)	(0.039)	(0.037)	(0.039)
*Public transportation pass holder*					
No (reference)					
Yes	0.068***	0.065***	0.060***	0.047***	0.061***
	(0.015)	(0.015)	(0.016)	(0.015)	(0.015)
No answer	0.116***	0.057	0.009	0.062*	0.054
	(0.039)	(0.040)	(0.038)	(0.036)	(0.038)
*Household monthly income*					
< CHF 900 (reference)					
CHF 901 - CHF 1,500	$-$0.046	0.060	$-$0.072	$-$0.003	$-$0.138**
	(0.055)	(0.059)	(0.059)	(0.053)	(0.061)
CHF 1,501 - CHF 2,000	0.046	0.124**	0.071	$-$0.009	0.027
	(0.054)	(0.058)	(0.054)	(0.052)	(0.054)
CHF 2,001 - CHF 3,000	0.048	0.147***	0.032	$-$0.001	0.059
	(0.047)	(0.051)	(0.048)	(0.045)	(0.047)
CHF 3,001 - CHF 4,000	$-$0.018	0.084*	$-$0.000	$-$0.033	0.008
	(0.044)	(0.049)	(0.045)	(0.043)	(0.045)
CHF 4,001 - CHF 5,000	$-$0.032	0.063	0.004	$-$0.036	$-$0.035
	(0.044)	(0.048)	(0.044)	(0.042)	(0.044)
CHF 5,001 - CHF 6,000	$-$0.060	0.063	$-$0.055	$-$0.017	$-$0.040
	(0.043)	(0.047)	(0.044)	(0.042)	(0.043)
CHF 6,001 - CHF 7,000	$-$0.066	0.104**	$-$0.022	$-$0.026	$-$0.036
	(0.045)	(0.048)	(0.045)	(0.043)	(0.045)
CHF 7,001 - CHF 8,000	$-$0.020	0.120**	0.021	$-$0.080*	0.010
	(0.046)	(0.049)	(0.046)	(0.045)	(0.046)
CHF 8,001 - CHF 9,000	$-$0.025	0.058	$-$0.050	$-$0.102**	$-$0.048
	(0.045)	(0.050)	(0.046)	(0.045)	(0.046)
CHF 9,001 - CHF 10,000	$-$0.020	0.096*	0.016	$-$0.063	$-$0.005
	(0.046)	(0.050)	(0.046)	(0.045)	(0.046)
CHF 10,001 - CHF 11,000	$-$0.023	0.046	$-$0.020	$-$0.061	$-$0.066
	(0.048)	(0.053)	(0.049)	(0.047)	(0.050)
CHF 11,001 - CHF 12,000	$-$0.096*	0.017	$-$0.048	$-$0.057	$-$0.062
	(0.052)	(0.056)	(0.052)	(0.049)	(0.051)
> CHF 12,000	$-$0.030	0.071	$-$0.018	$-$0.052	$-$0.020
	(0.043)	(0.048)	(0.044)	(0.042)	(0.044)
No answer	$-$0.053	0.051	$-$0.047	$-$0.046	$-$0.036
	(0.039)	(0.044)	(0.040)	(0.038)	(0.040)
*Education level*					
Compulsory schooling (reference)					
Apprenticeship	$-$0.004	$-$0.035	$-$0.031	$-$0.038	0.021
	(0.027)	(0.027)	(0.027)	(0.025)	(0.028)
Post-compulsory school	0.001	$-$0.020	$-$0.008	$-$0.020	0.035
	(0.026)	(0.026)	(0.026)	(0.024)	(0.026)
Superior first cycle degree	$-$0.032	0.006	$-$0.046	0.006	0.031
	(0.030)	(0.030)	(0.030)	(0.028)	(0.031)
Superior second cycle degree	$-$0.009	$-$0.028	$-$0.022	$-$0.009	0.034
	(0.026)	(0.026)	(0.026)	(0.024)	(0.026)
No answer	$-$0.052	$-$0.199**	$-$0.061	$-$0.000	$-$0.010
	(0.064)	(0.079)	(0.066)	(0.059)	(0.061)
*Residence area*					
Canton of Geneva (reference)					
Canton of Vaud	$-$0.067***	$-$0.024	$-$0.032	0.024	$-$0.048**
	(0.019)	(0.019)	(0.019)	(0.018)	(0.019)
Genevois	$-$0.033	$-$0.015	0.019	0.006	$-$0.003
	(0.034)	(0.032)	(0.032)	(0.031)	(0.032)
Gex	$-$0.029	$-$0.057***	$-$0.016	$-$0.001	0.005
	(0.021)	(0.021)	(0.020)	(0.019)	(0.020)
Haute-Savoie	0.040	0.073**	0.027	$-$0.003	0.017
	(0.035)	(0.034)	(0.035)	(0.034)	(0.036)
Annemasse agglomeration	0.025	0.040*	0.001	0.041**	$-$0.014
	(0.022)	(0.022)	(0.023)	(0.021)	(0.022)
*Commuting*					
Car (reference)					
Bus and tramway	0.015	0.009	0.018	0.019	0.013
	(0.020)	(0.020)	(0.021)	(0.019)	(0.020)
Train	0.055*	$-$0.010	0.011	$-$0.039	0.022
	(0.028)	(0.029)	(0.029)	(0.027)	(0.028)
Motorcycle	$-$0.006	0.015	$-$0.011	0.023	0.009
	(0.029)	(0.028)	(0.029)	(0.026)	(0.028)
Bicycle	0.068**	0.100***	0.037	$-$0.026	$-$0.010
	(0.032)	(0.032)	(0.033)	(0.034)	(0.034)
Walking	0.006	0.020	0.047	$-$0.009	0.029
	(0.031)	(0.031)	(0.030)	(0.030)	(0.030)
No answer	0.008	0.023	$-$0.001	$-$0.011	0.003
	(0.020)	(0.020)	(0.020)	(0.019)	(0.020)
Number of respondents	1414	1414	1414	1414	1414
Number of observations	5’638	5’678	5’638	5’651	5’651
$R^{2}$	0.0226	0.0224	0.0187	0.0131	0.0204

Estimates report average marginal effects from logit with control variables

Heteroskedasticity-robust standard errors in parentheses

* $$p<0.1$$, ** $$p<0.05$$, *** $$p<0.01$$

### Heterogeneity analysis results

**Table 19:** Latent classes: estimates from conditional logit

	(1)		(2)		(3)		(4)		(5)	
	Latent class 1		Latent class 2		Latent class 3		Latent class 4		Latent class 5	
*Charge rate*										
CHF 0 (reference)										
CHF 0.2	0.323***	(0.012)	$-$0.302***	(0.041)	0.069**	(0.032)	$-$0.316***	(0.051)	0.472***	(0.039)
CHF 1	0.334***	(0.013)	$-$0.236***	(0.042)	$-$0.179***	(0.031)	$-$0.332***	(0.053)	0.354***	(0.038)
CHF 2	0.319***	(0.013)	$-$0.225***	(0.039)	$-$0.376***	(0.036)	$-$0.507***	(0.077)	0.058	(0.038)
CHF 3	0.307***	(0.012)	$-$0.299***	(0.040)	$-$0.456***	(0.035)	$-$0.382***	(0.061)	$-$0.126***	(0.039)
CHF 4	0.297***	(0.013)	$-$0.274***	(0.042)	$-$0.469***	(0.036)	$-$0.439***	(0.055)	$-$0.290***	(0.039)
CHF 5	0.279***	(0.013)	$-$0.297***	(0.043)	$-$0.647***	(0.051)	$-$0.416***	(0.066)	$-$0.475***	(0.041)
*Perimeter*										
Center (reference)										
Ring	0.0004	(0.006)	$-$0.062**	(0.022)	0.010	(0.015)	$-$0.042	(0.028)	$-$0.029	(0.018)
*Modulation*										
Constant (reference)										
Peak hours only	0.010	(0.008)	$-$0.013	(0.023)	0.040*	(0.023)	0.054	(0.039)	0.001	(0.027)
Peak hours top-up	0.016**	(0.007)	0.005	(0.023)	$-$0.017	(0.023)	0.003	(0.043)	$-$0.027	(0.026)
Distance top-up	$-$0.028***	(0.008)	$-$0.036	(0.026)	$-$0.064**	(0.026)	$-$0.051	(0.050)	$-$0.082***	(0.028)
Pollution top-up	$-$0.001	(0.007)	0.008	(0.024)	$-$0.034	(0.022)	0.034	(0.040)	$-$0.061**	(0.026)
*Beneficiaries*										
Business deliveries (reference)										
Residents	0.021***	(0.008)	0.115***	(0.028)	$-$0.031	(0.022)	$-$0.002	(0.036)	$-$0.014	(0.028)
Motorbikes	$-$0.005	(0.008)	0.04	(0.030)	$-$0.066**	(0.026)	$-$0.039	(0.04)	$-$0.090***	(0.026)
Frequent commuters	0.014*	(0.008)	0.066**	(0.028)	$-$0.039*	(0.023)	0.043	(0.034)	$-$0.01	(0.026)
Electric vehicles	$-$0.007	(0.008)	0.081***	(0.027)	$-$0.132***	(0.025)	$-$0.208**	(0.092)	$-$0.106***	(0.028)
*Exemption level*										
0% (reference)										
25%	$-$0.005	(0.008)	0.011	(0.023)	0.043*	(0.024)	$-$0.116**	(0.051)	0.045*	(0.024)
50%	0.014*	(0.008)	0.047*	(0.024)	0.102***	(0.025)	$-$0.045	(0.037)	0.111***	(0.025)
75%	0.014*	(0.008)	0.062**	(0.025)	0.092***	(0.024)	$-$0.071	(0.040)	0.148***	(0.026)
100%	0.026***	(0.009)	0.092***	(0.026)	0.156***	(0.026)	$-$0.01	(0.033)	0.179***	(0.027)
*Use of revenue*										
Vehicle registration fee (reference)										
Public transportation	0.036***	(0.009)	0.247***	(0.027)	0.037	(0.023)	$-$0.038	(0.043)	0.038	(0.027)
Transport infrastructure	0.033***	(0.008)	0.122***	(0.029)	0.036	(0.023)	$-$0.012	(0.040)	0.020	(0.027)
Pollution reduction	0.021***	(0.008)	0.191***	(0.027)	$-$0.006	(0.022)	0.025	(0.037)	0.007	(0.027)
Tunnel or bridge	0.012	(0.008)	0.078**	(0.031)	0.030	(0.025)	$-$0.009	(0.038)	0.085***	(0.030)
Number of respondents	393		222		233		365		201	
Number of observations	11,784		6’660		6,990		10,947		6027	
Pseudo $R^{2}$	0.3028		0.0499		0.4381		0.9333		0.4550	

Estimates report average marginal effects from conditional logit

Heteroscedasticity-robust standard errors in parentheses

Continuous *p*-values are provided in Table 28

*$$p<0.01$$, **$$p<0.05$$, ***$$p<0.01$$

**Table 20:** Latent classes: membership estimates from multinomial logit

	(1)		(2)		(3)		(4)		(5)	
	Latent class 1		Latent class 2		Latent class 3		Latent class 4		Latent class 5	
< 30 years old	0.029	(0.034)	$-$0.014	(0.029)	0.01	(0.030)	$-$0.06*	(0.035)	0.03	(0.027)
> 65 years old	0.120	(0.208)	$-$2.091***	(0.157)	$-$2.270***	(0.161)	0.066	(0.206)	0.081	(0.129)
Gender (female)	0.016	(0.029)	0.024	(0.024)	0.003	(0.024)	$-$0.014	(0.028)	$-$0.028	(0.022)
Swiss	0.089**	(0.036)	0.034	(0.029)	0.002	(0.028)	0.067**	(0.032)	$-$0.171***	(0.026)
Resident of the center	0.058*	(0.034)	$-$0.023	(0.028)	$-$0.010	(0.032)	$-$0.048	(0.036)	0.023	(0.032)
Household size	0.016*	(0.008)	$-$0.002	(0.007)	$-$0.003	(0.007)	$-$0.026***	(0.009)	0.014**	(0.006)
Car and motorbike commuters	$-$0.059*	(0.030)	$-$0.072***	(0.025)	0.076***	(0.027)	0.092***	(0.03)	$-$0.037	(0.025)
Number of observations	29,940		29,940		29,940		29,940		29,940	
Number of respondents	998		998		9,98		998		998	
Pseudo $R^{2}$	0.0267		0.0187		0.0122		0.0265		0.0716	
Share of respondents	27.5%		16.3%		16.3%		25.7%		14.2%	

Estimates report average marginal effects

Heteroscedasticity-robust standard errors in parentheses

Continuous *p*-values are provided in Table 29

*$$p<0.05$$, **$$p<0.01$$, ***$$p<0.001$$

### Tables with *p*-values

**Table 21:** Estimates from conditional logit with *p*-values

	(1)		(2)		(3)	
	Full sample		Potential voters		Non-voters	
*Charge rate*						
CHF 0 (reference)						
CHF 0.2	$-$0.051***	(0.002)	$-$0.065***	(0.005)	$-$0.034	(0.167)
CHF 1	$-$0.109***	(0.000)	$-$0.099***	(0.000)	$-$0.119***	(0.000)
CHF 2	$-$0.195***	(0.000)	$-$0.173***	(0.000)	$-$0.22***	(0.000)
CHF 3	$-$0.262***	(0.000)	$-$0.232***	(0.000)	$-$0.296***	(0.000)
CHF 4	$-$0.283***	(0.000)	$-$0.239***	(0.000)	$-$0.34***	(0.000)
CHF 5	$-$0.33***	(0.000)	$-$0.291***	(0.000)	$-$0.375***	(0.000)
*Perimeter*						
Center (reference)						
Ring	$-$0.008	(0.263)	0.0001	(0.989)	$-$0.02*	(0.072)
*Modulation*						
Constant (reference)						
Peak hours only	0.018**	(0.042)	0.009	(0.458)	0.029**	(0.032)
Peak hours top-up	0.008	(0.395)	$-$0.0003	(0.983)	0.017	(0.205)
Distance top-up	$-$0.045***	(0.000)	$-$0.073***	(0.000)	$-$0.009	(0.542)
Pollution top-up	$-$0.008	(0.353)	$-$0.016	(0.193)	0.001	(0.945)
*Exemption level*						
0% (reference)						
25%	0.002	(0.839)	0.0004	(0.976)	0.004	(0.756)
50%	0.036***	(0.000)	0.036***	(0.004)	0.036**	(0.012)
75%	0.044***	(0.000)	0.041***	(0.002)	0.049***	(0.000)
100%	0.069***	(0.000)	0.046***	(0.000)	0.098***	(0.000)
*Beneficiaries*						
Business deliveries (reference)						
Residents	0.029***	(0.003)	0.060***	(0.000)	$-$0.014	(0.315)
Motorbikes	$-$0.015	(0.131)	$-$0.001	(0.923)	$-$0.035**	(0.019)
Frequent commuters	0.016*	(0.097)	0.022*	(0.085)	0.006	(0.653)
Electric vehicles	$-$0.023**	(0.022)	$-$0.003	(0.834)	$-$0.05***	(0.002)
*Use of revenue*						
Vehicle registration fee(reference)						
Public transportation	0.082***	(0.000)	0.084***	(0.000)	0.080***	(0.000)
Transport infrastructure	0.050***	(0.000)	0.056***	(0.000)	0.044***	(0.003)
Pollution reduction	0.051***	(0.000)	0.062***	(0.000)	0.039**	(0.011)
Tunnel or bridge	0.035***	(0.001)	0.025*	(0.067)	0.05***	(0.001)
Number of respondents	1,414		782		632	
Number of observations	42,408		23,454		18,954	
Pseudo $R^{2}$	0.0748		0.0523		0.1148	

Estimates report average marginal effects from conditional logit

*p*-values in parentheses

*$$p<0.1$$, **$$p<0.05$$, ***$$p<0.01$$

**Table 22:** The impact of informational treatments on public support, for each charge rate with *p*-values

	(1)	(2)	(3)	(4)	(5)	(6)
	CHF 0.2	CHF 1	CHF 2	CHF 3	CHF 4	CHF 5
Congestion	0.020	0.020	$-$0.003	0.002	$-$0.001	0.012
	(0.243)	(0.246)	(0.838)	(0.898)	(0.950)	(0.383)
Pollution	0.002	0.012	0.042**	0.037**	0.042***	0.038***
	(0.921)	(0.476)	(0.007)	(0.010)	(0.002)	(0.003)
Number of respondents	1414	1414	1414	1414	1414	1414
Number of observations	4,736	4,711	4,702	4,702	4,711	4,710
$R^{2}$	0.0003	0.0002	0.0019	0.0017	0.0027	0.0021

Estimates report average marginal effects from logit

*p*-values in parentheses

** $$p<0.05$$, *** $$p<0.01$$

**Table 23:** The impact of informational treatments on public support, for each modulation with *p*-values

	(1)	(2)	(3)	(4)	(5)
	Constant	Peak hours only	Peak hours top-up	Distance top-up	Pollution top-up
Congestion	0.019	$-$0.007	0.001	0.025	0.006
	(0.184)	(0.617)	(0.962)	(0.068)	(0.696)
Pollution	0.018	0.017	0.046***	0.022	0.045***
	(0.213)	(0.234)	(0.001)	(0.105)	(0.002)
Number of respondents	1414	1414	1414	1414	1414
Number of observations	5’638	5’678	5’646	5’659	5’651
$R^{2}$	0.0003	0.0004	0.002	0.0007	0.0018

Estimates report average marginal effects from logit

*p*-values in parentheses

*** $$p<0.01$$

**Table 24:** Estimates from conditional logit with *p*-values: residents of the perimeter, living in the Canton of Geneva but outside the perimeters, not living in the Canton of Geneva

	(1)		(2)		(3)	
	Residents		Living in the Canton of Geneva but outside the perimeters		Not living in the Canton of Geneva	
*Charge rate*						
CHF 0 (reference)						
CHF 0.2	$-$0.053*	(0.059)	$-$0.088**	(0.037)	$-$0.034	(0.167)
CHF 1	$-$0.072**	(0.012)	$-$0.150***	(0.000)	$-$0.119***	(0.000)
CHF 2	$-$0.144***	(0.000)	$-$0.231***	(0.000)	$-$0.220***	(0.000)
CHF 3	$-$0.215***	(0.000)	$-$0.264***	(0.000)	$-$0.296***	(0.000)
CHF 4	$-$0.210***	(0.000)	$-$0.302***	(0.000)	$-$0.340***	(0.000)
CHF 5	$-$0.268***	(0.000)	$-$0.336***	(0.000)	$-$0.375***	(0.000)
*Perimeter*						
Center (reference)						
Ring	$-$0.005	(0.680)	$-$0.008	(0.685)	$-$0.02*	(0.072)
*Modulation*						
Constant (reference)						
Peak hours only	$-$0.002	(0.898)	0.034	(0.156)	0.029**	(0.032)
Peak hours top-up	$-$0.018	(0.212)	0.041*	(0.065)	0.017	(0.205)
Distance top-up	$-$0.081***	(0.000)	$-$0.056**	(0.017)	$-$0.009	(0.542)
Pollution top-up	$-$0.036**	(0.011)	0.032	(0.140)	0.001	(0.945)
*Beneficiaries*						
Business deliveries (reference)						
Residents	0.068***	(0.000)	0.045*	(0.059)	$-$0.014	(0.315)
Motorbikes	$-$0.006	(0.712)	0.010	(0.676)	$-$0.035**	(0.019)
Frequent commuters	0.021	(0.183)	0.027	(0.272)	0.006	(0.653)
Electric vehicles	0.012	(0.453)	$-$0.037	(0.158)	$-$0.05***	(0.002)
*Exemption level*						
0% (reference)						
25%	0.01	(0.545)	$-$0.020	(0.344)	0.004	(0.756)
50%	0.039***	(0.010)	0.029	(0.184)	0.036**	(0.012)
75%	0.039**	(0.016)	0.048**	(0.021)	0.049***	(0.000)
100%	0.054***	(0.001)	0.026	(0.255)	0.098***	(0.000)
*Use of revenue*						
Vehicle registration fee (reference)						
Public transportation	0.087***	(0.000)	0.079***	(0.001)	0.080***	(0.000)
Transport infrastructure	0.058***	(0.000)	0.052**	(0.025)	0.044***	(0.003)
Pollution reduction	0.066***	(0.000)	0.053**	(0.026)	0.039**	(0.011)
Tunnel or bridge	0.020	(0.208)	0.038	(0.152)	0.050***	(0.001)
Number of respondents	547		235		632	
Number of observations	16,407		7,047		18,954	
Pseudo $R^{2}$	0.0456		0.0775		0.1148	

Estimates report average marginal effects from conditional logit

*p*-values in parentheses

*$$p<0.1$$, **$$p<0.05$$, ***$$p<0.01$$

**Table 25:** Estimates from conditional logit by commuting mode with *p*-values

	(1)		(2)	
	Car and motorbike		Public transportation, cycling and walking	
*Charge rate*				
CHF 0 (reference)				
CHF 0.2	$-$0.043	(0.110)	$-$0.056**	(0.010)
CHF 1	$-$0.140***	(0.000)	$-$0.086***	(0.000)
CHF 2	$-$0.248***	(0.000)	$-$0.159***	(0.000)
CHF 3	$-$0.321***	(0.000)	$-$0.224***	(0.000)
CHF 4	$-$0.328***	(0.000)	$-$0.252***	(0.000)
CHF 5	$-$0.388***	(0.027)	$-$0.292***	(0.000)
*Perimeter*				
Center (reference)				
Ring	$-$0.018	(0.138)	$-$0.004	(0.693)
*Modulation*				
Constant (reference)				
Peak hours only	0.031**	(0.041)	0.010	(0.352)
Peak hours top-up	0.014	(0.346)	0.004	(0.719)
Distance top-up	$-$0.012	(0.456)	$-$0.063***	(0.000)
Pollution top-up	0.004	(0.789)	$-$0.017	(0.142)
*Beneficiaries*				
Business deliveries (reference)				
Residents	$-$0.006	(0.723)	0.047***	(0.000)
Motorbikes	$-$0.016	(0.334)	$-$0.017	(0.193)
Frequent commuters	0.009	(0.559)	0.018	(0.132)
Electric vehicles	$-$0.067***	(0.000)	0.002	(0.896)
*Exemption level*				
0% (reference)				
25%	0.020	(0.190)	$-$0.007	(0.537)
50%	0.045***	(0.004)	0.031***	(0.009)
75%	0.061***	(0.000)	0.036***	(0.003)
100%	0.096***	(0.000)	0.054***	(0.000)
*Use of revenue*				
Vehicle registration fee (reference)				
Public transportation	0.061***	(0.000)	0.093***	(0.000)
Transport infrastructure	0.033**	(0.035)	0.062***	(0.000)
Pollution reduction	0.051***	(0.001)	0.052***	(0.000)
Tunnel or bridge	0.05***	(0.002)	0.027**	(0.042)
Number of respondents	544		870	
Number of observations	16,314		26,094	
Pseudo $R^{2}$	0.1206		0.0552	

Estimates report average marginal effects from conditional logit

*p*-values in parentheses

*$$p<0.1$$, **$$p<0.05$$, ***$$p<0.01$$

**Table 26:** Estimates from conditional logit by commuting frequency with *p*-values

	(1)		(2)		(3)	
	Inhabitants of the Canton of Geneva		6-7 trips/week to Geneva		1-5 trips/week to Geneva	
*Charge rate*						
CHF 0 (reference)						
CHF 0.2	$-$0.046	(0.104)	$-$0.103***	(0.005)	$-$0.03	(0.246)
CHF 1	$-$0.0742***	(0.009)	$-$0.151***	(0.000)	$-$0.119***	(0.000)
CHF 2	$-$0.151***	(0.000)	$-$0.255***	(0.000)	$-$0.201***	(0.000)
CHF 3	$-$0.214***	(0.000)	$-$0.288***	(0.000)	$-$0.290***	(0.000)
CHF 4	$-$0.205***	(0.000)	$-$0.322***	(0.000)	$-$0.335***	(0.000)
CHF 5	$-$0.266***	(0.000)	$-$0.340***	(0.000)	$-$0.383***	(0.000)
*Perimeter*						
Center (reference)						
Ring	0.002	(0.861)	0.006	(0.696)	$-$0.026**	(0.019)
*Modulation*						
Constant (reference)						
Peak hours only	$-$0.008	(0.582)	0.038**	(0.048)	0.031**	(0.032)
Peak hours top-up	$-$0.01	(0.506)	0.017	(0.388)	0.02	(0.162)
Distance top-up	$-$0.084***	(0.000)	$-$0.055***	(0.008)	$-$0.004	(0.804)
Pollution top-up	$-$0.024*	(0.099)	$-$0.019	(0.364)	0.013	(0.331)
*Beneficiaries*						
Business deliveries (reference)						
Residents	0.053***	(0.001)	0.031	(0.117)	0.003	(0.820)
Motorbikes	$-$0.009	(0.604)	0.013	(0.533)	$-$0.04**	(0.010)
Frequent commuters	0.021	(0.192)	0.041**	(0.048)	$-$0.003	(0.826)
Electric vehicles	$-$0.011	(0.501)	$-$0.007	(0.732)	$-$0.043**	(0.010)
*Exemption level*						
0% (reference)						
25%	$-$0.001	(0.958)	$-$0.0002	(0.990)	0.007	(0.628)
50%	0.025	(0.108)	0.042**	(0.023)	0.043***	(0.005)
75%	0.026	(0.110)	0.065***	(0.002)	0.050***	(0.000)
100%	0.041**	(0.011)	0.045**	(0.030)	0.107***	(0.000)
*Use of revenue*						
Vehicle registration fee (reference)						
Public transportation	0.076***	(0.000)	0.106***	(0.000)	0.075***	(0.000)
Transport infrastructure	0.045***	(0.003)	0.060***	(0.007)	0.051***	(0.001)
Pollution reduction	0.054***	(0.001)	0.062***	(0.005)	0.044***	(0.007)
Tunnel or bridge	0.007	(0.688)	0.054**	(0.018)	0.052***	(0.002)
Number of respondents	515		314		585	
Number of observations	15,444		9,414		17,550	
Pseudo $R^{2}$	0.0531		0.0735		0.1057	

Estimates report average marginal effects from conditional logit

*p*-values in parentheses

*$$p<0.1$$, **$$p<0.05$$, ***$$p<0.01$$

**Table 27:** Estimates from conditional logit with *p*-values: cars vs. motorbikes

	(1)		(2)	
	Car		Motorbike	
*Charge rate*				
CHF 0 (reference)				
CHF 0.2	$-$0.022	(0.445)	$-$0.159**	(0.021)
CHF 1	$-$0.124***	(0.000)	$-$0.234***	(0.001)
CHF 2	$-$0.233***	(0.000)	$-$0.334***	(0.000)
CHF 3	$-$0.319***	(0.000)	$-$0.343***	(0.000)
CHF 4	$-$0.321***	(0.000)	$-$0.376***	(0.000)
CHF 5	$-$0.375***	(0.000)	$-$0.454***	(0.000)
*Perimeter*				
Center (reference)				
Ring	$-$0.02	(0.117)	$-$0.008	(0.794)
*Modulation*				
Constant (reference)				
Peak hours only	0.029*	(0.081)	0.05	(0.174)
Peak hours top-up	0.013	(0.422)	0.028	(0.463)
Distance top-up	$-$0.013	(0.448)	0.008	(0.838)
Pollution top-up	0.004	(0.820)	0.013	(0.733)
*Beneficiaries*				
Business deliveries (reference)				
Resident	$-$0.004	(0.796)	$-$0.008	(0.808)
Motorbikes	$-$0.048***	(0.006)	0.147***	(0.000)
Frequent commuters	0.013	(0.446)	$-$0.019	(0.614)
Electric vehicles	$-$0.073***	(0.000)	$-$0.026	(0.514)
*Exemption level*				
0% (reference)				
25%	0.011	(0.503)	0.071*	(0.075)
50%	0.037**	(0.025)	0.081**	(0.040)
75%	0.052***	(0.002)	0.098***	(0.004)
100%	0.092***	(0.000)	0.117***	(0.002)
*Use of revenue*				
Vehicle registration fee (reference)				
Public transportation	0.053***	(0.002)	0.101***	(0.007)
Transport infrastructure	0.036**	(0.031)	0.009	(0.828)
Pollution reduction	0.054***	(0.001)	0.029	(0.477)
Tunnel or bridge	0.045**	(0.011)	0.085**	(0.047)
Number of respondents	465		79	
Number of observations	13,944		2,370	
Pseudo $R^{2}$	0.1285		0.1066	

Estimates report average marginal effects from conditional logit

*p*-values in parentheses

*$$p<0.1$$, **$$p<0.05$$, ***$$p<0.01$$

**Table 28:** Latent classes: estimates from conditional logit with *p*-values

	(1)		(2)		(3)		(4)		(5)	
	Latent class 1		Latent class 2		Latent class 3		Latent class 4		Latent class 5	
*Charge rate*										
CHF 0 (reference)										
CHF 0.2	0.323***	(0.000)	$-$0.302***	(0.000)	0.069**	(0.031)	$-$0.316***	(0.000)	0.472***	(0.000)
CHF 1	0.334***	(0.000)	$-$0.236***	(0.000)	$-$0.179***	(0.000)	$-$0.332***	(0.000)	0.354***	(0.000)
CHF 2	0.319***	(0.000)	$-$0.225***	(0.000)	$-$0.376***	(0.000)	$-$0.507***	(0.000)	0.058	(0.130)
CHF 3	0.307***	(0.000)	$-$0.299***	(0.000)	$-$0.456***	(0.000)	$-$0.382***	(0.000)	$-$0.126***	(0.001)
CHF 4	0.297***	(0.000)	$-$0.274***	(0.000)	$-$0.469***	(0.000)	$-$0.439***	(0.000)	$-$0.290***	(0.000)
CHF 5	0.279***	(0.000)	$-$0.297***	(0.000)	$-$0.647***	(0.000)	$-$0.416***	(0.000)	$-$0.475***	(0.000)
*Perimeter*										
Center (reference)										
Ring	0.0004	(0.944)	$-$0.062***	(0.005)	0.010	(0.499)	$-$0.042	(0.137)	$-$0.029	(0.117)
*Modulation*										
Constant (reference)										
Peak hours only	0.010	(0.204)	$-$0.013	(0.583)	0.040*	(0.084)	0.054	(0.164)	0.001	(0.976)
Peak hours top-up	0.016**	(0.031)	0.005	(0.832)	$-$0.017	(0.475)	0.003	(0.946)	$-$0.027	(0.304)
Distance top-up	$-$0.028***	(0.000)	$-$0.036	(0.163)	$-$0.064**	(0.013)	$-$0.051	(0.315)	$-$0.082***	(0.003)
Pollution top-up	$-$0.001	(0.924)	0.008	(0.750)	$-$0.034	(0.127)	0.034	(0.407)	$-$0.061**	(0.020)
*Beneficiaries*										
Business deliveries (reference)										
Residents	0.021***	(0.008)	0.115***	(0.000)	$-$0.031	(0.161)	$-$0.002	(0.963)	$-$0.014	(0.609)
Motorbikes	$-$0.005	(0.512)	0.04	(0.191)	$-$0.066**	(0.012)	$-$0.039	(0.322)	$-$0.090***	(0.001)
Frequent commuters	0.014*	(0.074)	0.066**	(0.018)	$-$0.039*	(0.087)	0.043	(0.208)	$-$0.01	(0.712)
Electric vehicles	$-$0.007	(0.358)	0.081***	(0.003)	$-$0.132***	(0.000)	$-$0.208**	(0.024)	$-$0.106***	(0.000)
*Exemption level*										
0% (reference)										
25%	$-$0.005	(0.552)	0.011	(0.627)	0.043*	(0.075)	$-$0.116**	(0.022)	0.045*	(0.063)
50%	0.014*	(0.075)	0.047*	(0.057)	0.102***	(0.000)	$-$0.045	(0.222)	0.111***	(0.000)
75%	0.014*	(0.084)	0.062**	(0.014)	0.092***	(0.000)	$-$0.071	(0.079)	0.148***	(0.000)
100%	0.026***	(0.002)	0.092***	(0.000)	0.156***	(0.000)	$-$0.01	(0.763)	0.179***	(0.000)
*Use of revenue*										
Vehicle registration fee (reference)										
Public transportation	0.036***	(0.000)	0.247***	(0.000)	0.037	(0.105)	$-$0.038	(0.373)	0.038	(0.159)
Transport infrastructure	0.033***	(0.000)	0.122***	(0.000)	0.036	(0.120)	$-$0.012	(0.760)	0.020	(0.4537)
Pollution reduction	0.021***	(0.010)	0.191***	(0.000)	$-$0.006	(0.773)	0.025	(0.494)	0.007	(0.791)
Tunnel or bridge	0.012	(0.142)	0.078**	(0.012)	0.030	(0.230)	$-$0.009	(0.818)	0.085***	(0.005)
Number of respondents	393		222		233		365		201	
Number of observations	11,784		6’660		6,990		10,947		6027	
Pseudo $R^{2}$	0.3028		0.0499		0.4381		0.9333		0.4550	

Estimates report average marginal effects from conditional logit

*p*-values in parentheses

* $$p<0.01$$, ** $$p<0.05$$, *** $$p<0.01$$

**Table 29:** Latent classes: membership estimates from multinomial logit with *p*-values

	(1)		(2)		(3)		(4)		(5)	
	Latent class 1		Latent class 2		Latent class 3		Latent class 4		Latent class 5	
< 30 years old	0.029	(0.404)	$-$0.014	(0.629)	0.014	(0.634)	$-$0.064*	(0.070)	0.03	(0.277)
> 65 years old	0.120	(0.565)	$-$2.091***	(0.000)	$-$2.270***	(0.000)	0.066	(0.748)	0.081	(0.529)
Gender (female)	0.016	(0.579)	0.024	(0.314)	0.003	(0.896)	$-$0.014	(0.603)	$-$0.028	(0.205)
Swiss	0.089**	(0.014)	0.040	(0.178)	0.002	(0.945)	0.067**	(0.037)	$-$0.171***	(0.000)
Resident of the center	0.058*	(0.084)	$-$0.023	(0.421)	$-$0.010	(0.751)	$-$0.048	(0.182)	0.023	(0.477)
Household size	0.016*	(0.050)	$-$0.002	(0.753)	$-$0.003	(0.632)	$-$0.026***	(0.004)	0.014**	(0.022)
Car and motorbike commuters	$-$0.059*	(0.051)	$-$0.072***	(0.004)	0.076***	(0.004)	0.092***	(0.002)	$-$0.037	(0.134)
Number of observations	29,940		29,940		29,940		29,940		29,940	
Number of respondents	998		998		9,98		998		998	
Pseudo $R^{2}$	0.0267		0.0187		0.0122		0.0265		0.0716	
Share of respondents	27.5%		16.3%		16.3%		25.7%		14.2%	

Estimates report average marginal effects

*p*-values in parentheses

* $$p<0.05$$, ** $$p<0.01$$, *** $$p<0.001$$

## Footnotes

1 1CHF $\approx$ 1USD at the time of the study.

2 See https://www.tomtom.com/ (last accessed on July 10, 2020) and https://inrix.com/scorecard (last accessed on July 10, 2020).

3 The questionnaire was pre-tested with about 300 individuals. Qualitative interviews had also been conducted, to inform the questionnaire.

4 The novel approach that we present in this paper combines a discrete choice experiment with randomized information provision about policy effectiveness. Other contexts in which information provision about policy effectiveness has been provided as a randomized treatment include split-sample surveys (e.g. Kaplowitz and McCright 2015) and lab experiments (e.g. Baranzini et al. 2018a). Unlike before-after comparisons (e.g. Rhodes et al. 2014), random assignment to treatment and control groups allows for clean identification and limits experimenter effects.

5 As per standard procedure, we minimize the Akaike information criterion (AIC) and the Bayesian information criterion (BIC). AIC (BIC) values are given as follows: for two classes, 23049 (23002); for three classes, 21902 (21831); for four classes, 21658 (21563); for five classes, 21602 (21483); for six classes, 21644 (21501).
